# High-resolution multiomics links nutrients and mixotrophy to toxicity in a harmful bloom of the haptophyte *Chrysochromulina leadbeateri*

**DOI:** 10.1126/sciadv.adv3390

**Published:** 2025-06-25

**Authors:** Antonia Otte, Sylke Wohlrab, Franco Moritz, Constanze Müller, Jan Janouškovec, Jan Michálek, Allan Cembella, Daniela Voss, Xinhui Wang, Jan Tebben, Thomas Ostenfeld Larsen, Bente Edvardsen, Philippe Schmitt-Kopplin, Uwe John

**Affiliations:** ^1^ Department of Ecological Chemistry, Alfred Wegener Institute (AWI), Helmholtz Centre for Polar and Marine Research, Bremerhaven, Germany.; ^2^ Helmholtz Institute for Functional Marine Biodiversity at the University of Oldenburg (HIFMB), Oldenburg, Germany.; ^3^ Analytical BioGeoChemistry, Helmholtz Zentrum München, German Research Center for Environmental Health, Neuherberg, Germany.; ^4^ Section for Aquatic Biology and Toxicology, Department of Biosciences, University of Oslo, Oslo, Norway.; ^5^ Centre Algatech, Institute of Microbiology of the Czech Academy of Sciences, Třeboň, Czech Republic.; ^6^ School of Biological Sciences, University of Southampton, Southampton, UK.; ^7^ Departamento de Biotecnología Marina, Centro de Investigación Científica y Educación Superior de Ensenada (CICESE), Baja California, Ensenada, Mexico.; ^8^ Institute for Chemistry and Biology of the Marine Environment (ICBM), University of Oldenburg, Oldenburg, Germany.; ^9^ Department of Biotechnology and Biomedicine, Technical University of Denmark, Lyngby, Denmark.; ^10^ Analytical Food Chemistry, Technical University of Munich, Freising, Germany.

## Abstract

Harmful algal blooms (HABs) of the toxigenic haptophyte *Chrysochromulina* are known to cause fish mortalities and collateral ecosystem damage. The ichthyotoxic mechanisms are poorly understood but likely dependent on toxigenesis by polyketide synthases (PKSs). We hypothesize that induction of PKS activity facilitates mixotrophic behavior during nutrient-depleted bloom conditions. To identify potential in situ stimuli for growth, toxigenicity, and bloom persistence, we compared environmental factors and biological processes identified by metaomics to *Chrysochromulina leadbeateri* HABs between two fjords in northern Norway. We identified the polyketide ichthyotoxin leadbeaterin-1 from the *C. leadbeateri* bloom and found potentially associated candidate PKS genes of which most were higher expressed at bloom stations. A relative depletion of inorganic nitrogen and phosphate during the bloom was correlated with higher expression of genes involved in endocytosis, autophagy, and lysosomal activity. Mixotrophy is evidently a compensatory nutritional strategy coupled to induction of toxigenesis and other metabolomic processes as biotic factors linked to *Chrysochromulina* bloom dynamics.

## Teaser

Linking environmental drivers with multiomics in a harmful algal bloom reveals survival strategies.

## INTRODUCTION

Eukaryotic microalgae (also known as protists) can dominate the plankton community with beneficial effects on marine food web dynamics but, in some cases, form harmful algal blooms (HABs) with diverse negative impacts on the functions and services of aquatic ecosystems (*1*). HABs cause severe effects on fisheries, aquaculture, tourism and recreation, human health, and other socioeconomic activities, primarily in coastal waters worldwide (*2*). On a global scale, even the most comprehensive meta-analysis of time-series data cannot successfully explain or predict the magnitude and frequency of HABs (*3*). On a regional scale, however, changes in biogeographical distribution and bloom dynamics have circumstantially been associated to anthropogenic activities, especially to aquaculture industries, coastal zone modifications, and human-induced climate change (*3*–*5*).

In northern Europe and along eastern North Atlantic marginal sea coasts, HABs have at times caused major socioeconomic damage [reviewed in (*6*, *7*)]. Episodic blooms over the past 50 years have resulted in major losses to the aquaculture and fishing industries, posed a risk to human health from consumption of toxic seafood, and disrupted local ecosystem functioning. Blooms of the haptophyte genera *Chrysochromulina* and *Prymnesium* have caused mass fish mortalities and other ecosystem disruptions worldwide; in northern Europe, these fish-killing events have been particularly prominent along the Norwegian coast and into the Skagerrak and Kattegat shared between western Sweden and Denmark, especially during the late 1980s and 1990s (*6*–*8*). During May to June 1988, for example, an extensive bloom of *Prymnesium polylepis* (Manton and Parke) Edvardsen, Eikrem, and Probert (previously known as *Chrysochromulina polylepis* Manton and Parke) occurred in the Skagerrak, Kattegat and along the eastern North Sea coast of Norway. This massive bloom killed not only farmed salmon but also wild fish and a wide range of benthic fauna (*9*). From 1989 to 1995, yearly blooms of *P. parvum* (Green, Hibberd, and Pienaar) caused mortalities of farmed fish in a fjord system in Ryfylke, western Norway (*10*). In May to June 1991, a third haptophyte, identified as *C. leadbeateri*, bloomed in northern Norway (Vestfjorden) causing high mortalities of farmed salmon (*11*). Then, after a gap of 28 years in major fish-killing events due to haptophytes, an unprecedented massive bloom of *C. leadbeateri* persisted from early May to June 2019 in northern Norwegian coastal waters (*12*). This singular HAB event caused death of >14,500 metric tons of aquaculture salmon in late May, corresponding to 2% of the Norwegian fisheries harvested biomass and causing socioeconomic losses of ~$300 million (*13*).

Causes for the formation and toxicity of haptophyte blooms are usually interpreted with respect to bottom-up abiotic factors (*8*, *11*, *12*), such as nutrient availability and supply ratios, coupled with physical factors including temperature, pH, salinity, degree of stratification, weather, and bloom advection. Oceanographic and microplankton biodiversity conditions of the 2019 bloom in northern Norway have been described from DNA metabarcoding and integrating physicochemical and biotic factors with the dynamics and distribution of the bloom (*12*). Such studies have, however, provided little insight into biotic factors, cellular processes, and metabolic responses to species interactions for bloom formation and maintenance.

Some haptophyte blooms are accompanied by mass production of polysaccharide mucus that can cause collateral harm to the local ecosystem. These blooms may also produce bioactive substances, including putative ichthyotoxins and membrane lytic compounds, that are circumstantially implicated in fish kills. Despite the fact that members of genus *Prymnesium* and phylogenetically related *Chrysochromulina* within the same order Prymnesiales are often generally considered to be “fish-killers,” not all species or strains are ichthyotoxic. Certain *Prymnesium*, including the former *C. polylepis*, can express similar cell lytic activity in bioassays with fish blood cells and gill tissue and, e.g., against the cryptomonad *Rhodomonas* (*14*–*17*). Lytic substances produced by ichthyotoxic *Chrysochromulina* and *Prymnesium* may act by disrupting cell membranes, presumably causing ichthyotoxicity via gill damage that increase cell membrane permeability and disturb the ion balance of cells [reviewed by (*8*)]. However, the exact mechanisms of haptophyte-mediated fish kills and the potential role of such ichthyotoxins in growth and bloom dynamics remain obscure.

One candidate group of plausible haptophyte ichthyotoxins includes multiple subclasses of ladder-frame polyethers known as prymnesins, produced by certain strains of *P. parvum* (*18*–*20*). Prymnesins are biosynthesized by the largest gene, transcript, and protein complex found so far (*20*) and are derived via similar polyketide synthase (PKS) pathways among strains (*21*). Several research groups have hypothesized production of PKS-derived ichthyotoxins similar if not identical to prymnesins by other haptophytes. Nevertheless, direct confirmation for PKS-derived ichthyotoxins in *Chrysochromulina* came only recently, with the discovery of a putative ichthyotoxic polyketide, leadbeaterin-1 (LBT-1), characterized from cell cultures of *C. leadbeateri* and later identified in natural blooms from northern Norway (*17*).

The biological factors responsible for *C. leadbeateri*–dominated blooms, specifically the relationships among putative ichthyotoxins, membrane-disruptive lytic substances against plankton competitors and grazers, and mixotrophy as a nutritional mode, remain poorly understood for all haptophytes. Many members of the haptophyte order Prymnesiales have been shown to be facultative mixotrophs able to ingest food particles, in some cases [e.g., *Haptolina* (*Chrysochromulina*) *hirta*] facilitated by the haptonema (*22*). Mixotrophic behavior in *C. leadbeateri* has also been specifically demonstrated, with evidence of phagotrophy shown via a food vacuole situated near the flagella and haptonema, from field bloom specimens from Norwegian coastal water (*23*). The lytic activity of ichthyotoxins may enhance mixotrophic prey capture by immobilization, followed by phagotrophy or pinocytosis of organic nutrients after prey cell lysis (*24*, *25*). These lytic substances could have further inducible allelopathic functions to favor *C. leadbeateri* in interspecific competition for nutrients (*26*, *27*) or by repelling grazers (*28*). In laboratory cultures of *P. parvum*, for example, low salinity and/or phosphate-limited growth can induce toxigenicity and increase cell toxin quotas (*15*, *29*, *30*). Such toxigenicity induction from nutrient manipulation studies on isolated bloom phenotypes, however, are difficult to interpret in the context of natural haptophyte blooms.

In this study, we applied high-throughput multiomics approaches (*31*) to allow linking cellular processes associated with cell proliferation and/or toxigenicity to environmental factors determined during an in situ ichthyotoxic bloom of *C. leadbeateri*. We analyzed the gene expression patterns (metatranscriptome) in conjunction with abiotic oceanographic factors (i.e., temperature and inorganic macronutrient concentrations) and biotic determinants, including metabolomic profiles and production of bioactive compounds (i.e., putative ichthyotoxins), to identify key factors affecting *C. leadbeateri* bloom toxicity and microbial community dynamics. We hypothesize that *C. leadbeateri* bloom formation and toxicity are initially triggered by abiotic environmental conditions but that the induced toxicity then facilitates mixotrophy to compensate for emerging macronutrient limitation to sustain dominance of the bloom phenotype. To explore this biotic scenario, we determined the gene expression profile of *C. leadbeateri* within a natural bloom assemblage in Balsfjorden, northern Norway, during a high cell density and dominant *C. leadbeateri* bloom, and contemporaneously compared with an abiotically similar fjord in northern Norway, but with low *C. leadbeateri* cell abundance. We confirmed toxigenicity of the *Chrysochromulina* bloom with a high-content screening (HCS) approach and the presence of the recently identified putative ichthyotoxin LBT-1 by high-resolution tandem mass spectrometry (HR-MS/MS). Gene expression profiles were then linked to identify PKS-candidate genes for polyketide biosynthesis, presumably including putative ichthyotoxins such as leadbeaterins (LBTs) and prymnesins known to be synthesized by various fish-killing haptophytes.

The alignment of bloom transcriptomic profiles with detailed metabolomic analyses allowed the development of an unprecedented functional scenario for a mixotrophic haptophyte bloom with toxigenic capacity. This conceptual model incorporates abiotic and biotic factors through successional stages and highlights mixotrophy as a key nutritional strategy for extended bloom development and maintenance.

## RESULTS

### Oceanographic parameters and environmental nutrient variables

**Fig. 1.**
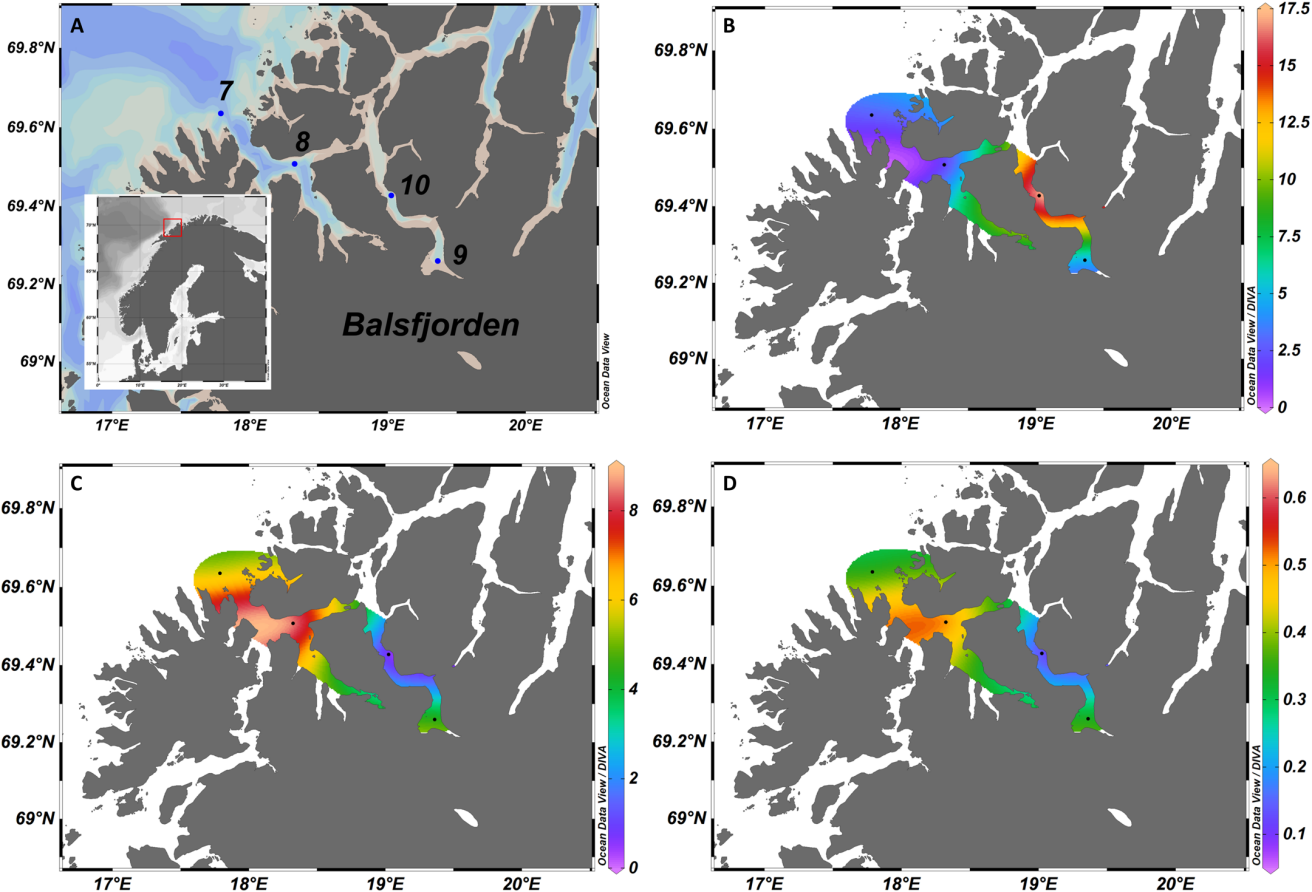
Map of the study area. Stations sampled in Balsfjorden (**A**) showing the spatial distribution of extracted chl a (μg liter^−1^) (**B**), total inorganic nitrogen (TIN) (μmol liter^−1^) (**C**) and inorganic phosphate (PO_4_^−3^) (μmol liter^−1^) (**D**) at the chlorophyll maximum layer.

The physical structure of the water column in both Balsfjorden and Porsangerfjorden systems was typical for these fjords during summer, with higher water temperature and lower salinity toward the inner part of the fjords, leading to similarly increased stratification of the surface layer (shown in vertical transects) (fig. S1). In contrast, a Pearson pairwise correlation matrix analysis (fig. S2) for multiple environmental parameters [longitude, depth at chlorophyll maximum layer, temperature, salinity, inorganic nitrogen (NO_2_^−^, NO_3_^−^, and NH_4_^+^), total N, inorganic phosphate (PO_4_^−3^), and N:P ratios] shows noteworthy differences between the fjord systems. The water temperature in Balsfjorden (5.7° to 8.8°C) was significantly higher than in the more northerly Porsangerfjorden (between 2.6° and 4.7°C); Pearson correlation coefficient (*r*) with longitude: *r* = −0.7, *P* = 0.05405; fig. S2). The salinity was overall higher at lower temperatures (*r* = −0.82, *P* < 0.05), but there were no highly significant correlations, e.g., between salinity or silicate (SiO_4_), with station longitude, or any other parameters (*r* < 0.65 and *P* > 0.1). Spatial distribution of total inorganic N (TIN; sum of NH_4_, NO_2_^−^, and NO_3_^−^) and inorganic P (PO_4_^−3^) throughout the fjords indicate a negative association to the chlorophyll (chl a) biomass (Fig. 1). In general, the lowest inorganic N and P nutrient concentrations were found toward the inner part of the respective fjord system in association with the highest chl a biomass [at station (St) 10].

In Balsfjorden, TIN and phosphate (PO_4_^−3^) concentrations were lower at stations 9 and 10, defined as “peak bloom” by cell densities, compared to (pre-bloom) stations 7 and 8 with lower *C. leadbeateri* cell densities. There was relatively less TIN at Balsfjorden stations compared to Porsangerfjorden, with lower N:P ratios most pronounced within the bloom at St 10. Regarding the total particulate organic nutrient ratios (POC:PON; POC:POP), POC:PON did not differ significantly (Pearson correlation) with longitude (*P* > 0.2), but the ratios POC:POP (*r* = −0.85; *P* = 0.015) and PON:POP (*r* = −0.9; *P* = 0.005) were significantly correlated with longitude and higher in Balsfjorden (fig. S3).

### Chlorophyll a biomass and *Chrysochromulina* cell density

For further analyses on selected parameters that may have greatest effect on the bloom dynamics and subsequent environmental impacts, we focused on the chlorophyll maximum layer, determined as an in vivo chlorophyll biomass proxy from the fluorescence sensor on the conductivity-temperature-depth (CTD) probe. The highest chl a biomass determined at the chlorophyll maximum layer from extracted chlorophyll from discrete samples occurred at St 10 (17.43 μg liter^−1^) and coincided with the bloom peak of *C. leadbeateri*. Decreasing chl a levels were determined at St 9 (4.76 μg liter^−1^) and St 7 (3.04 μg liter^−1^) and <3 μg liter^−1^ at all other stations (compare in table S1 and see corresponding maps for Porsangerfjorden in fig. S4). The highest *C. leadbeateri* cell densities were recorded at St 10 (2.7 × 10^7^ cells liter^−1^), followed by St 9 (2.0 × 10^7^), St 8 (2.4 × 10^6^), and St 7 (1.1 × 10^6^), and <1.3 × 10^4^ at all Porsangerfjorden stations (see fig. S5). Correlating values across all stations revealed that lower chl a biomass was associated with lower *C. leadbeateri* cell densities (Pearson correlation; *P* = 0.004) (table S1) and generally reflected the relative *Chrysochromulina* bloom dominance.

### Toxigenicity and putative ichthyotoxic metabolites

Cytotoxicity values of human osteosarcoma cell line U-2 OS normalized for *C. leadbeateri* cell numbers provided evidence for high cytotoxicity and the presence of putative ichthyotoxins in *Chrysochromulina*-dominated bloom samples from Balsfjorden. High-content screening (HCS) of human U-2 OS cell line for toxigenicity of field plankton samples collected from the *C. leadbeateri* bloom at Balsfjorden stations showed significantly stronger (*P* < 0.05) cytotoxic effects on specific organelles of U-2 OS cells compared with extracts from non-bloom stations in Porsangerfjorden (showed no effect) (fig. S6).

Analysis on two independent HR-MS/MS platforms confirmed that both the dissolved and particulate fractions collected from the chlorophyll maximum layer during the *Chrysochromulina* bloom in Balsfjorden yielded ion-spectra peaks consistent with an analyte with molecular formula C_67_H_127_Cl_1_O_27_. The ion fragmentation pattern, e.g., daughter ions *m/z* (mass/charge ratio) 725 and 423 (fig. S7 and table S2), and chromatographic retention time were both congruent with LBT-1. Other putative LBT analogs were also detected (e.g., C_67_H_124_Cl_2_O_27_, C_67_H_128_O_27_, and C_67_H_127_ClO_30_S), with the maximum abundance found at St 9 for all analogs (table S3).

The ratio of LBT-1 in the combined dissolved (solid-phase extracted) and particulate (GF/F filter-retained) fractions in Balsfjorden was 99:1 at St 9 and 7:3 at St 10, respectively. The concentration ratio of LBT-1 among Balsfjorden (bloom) stations (St 9 = 100:St 10 = 5) and Porsangerfjorden (non-bloom) stations (St 18 = 2) (methodological details in Supplementary Text) was generally consistent with the relative densities of *C. leadbeateri*–like cells. Nevertheless, there were anomalies—the highest concentration of LBT-1 was found at St 9 (20-fold higher than at St 10) but at a 35% lower *Chrysochromulina* cell density (table S1). On the basis of high *C. leadbeateri* cell numbers, high HCS-defined toxicity and elevated LBT-1 concentrations, we distinguished stations 9 and 10 as “peak-bloom” while referring to stations 7 and 8 as “pre-bloom.”

### Metatranscriptomic analysis of the microalgal bloom

Metatranscriptomic mapping against reference transcriptomes of *C. leadbeateri* showed high transcriptional activity (up to 58% of total sequence reads) of *C. leadbeateri* in Balsfjorden, whereas in Porsangerfjorden read counts attributable to this species were only <9% of total reads, roughly corresponding to its relative cell abundance. The number of total sequenced reads across Balsfjorden and Porsangerfjorden samples ranged from 36,912,472 (St 10B) to 58,355,532 (St 09C). Here, we distinguish St 9 and St 10 as peak bloom locations due to high sequence-read counts mapped to *C. leadbeateri* from both metabarcoding (*12*) and metatranscriptomic analyses (fig. S8).

**Fig. 2.**
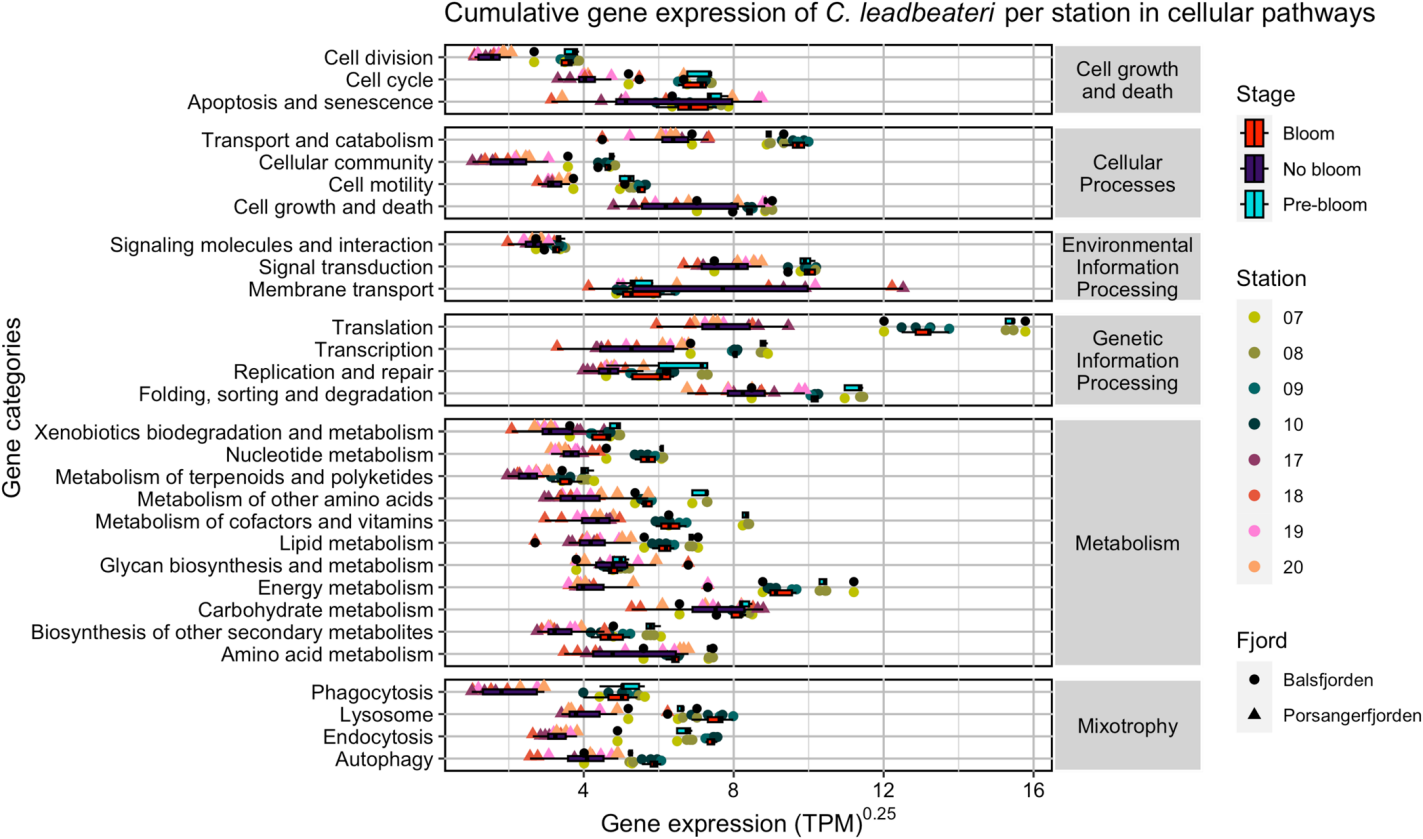
Normalized gene expression of *C. leadbeateri* in Balsfjorden and Porsangerfjorden. Gene expression is shown in TPM^0.25^. Genes annotated and assigned to KEGG categories are grouped according to KEGG pathways level 1 (right-hand side: “Environmental Information Processing,” “Genetic Information Processing,” “Metabolism,” and “Cellular processes”) or grouped as genes putatively involved in “Cell growth and death” or “Mixotrophy.” For a list of genes included in the categories, see table S4. Colors represent values obtained from the stations. Box plots show differences among Balsfjorden bloom St 9 and St 10; pre-bloom Balsfjorden St 7 and St 8; and no bloom Porsangerfjorden St 17 to St 20.

Comparing relative abundance of *C. leadbeateri* genes based on the Kyoto Encyclopedia of Genes and Genomes (KEGG) pathway annotation revealed variation within and differences between the studied fjord systems (Fig. 2). We found substantial differences in expressed KEGG pathways between Balsfjorden and Porsangerfjorden, in particular for the KEGG categories Energy Metabolism, Cellular Community, Transcription, Translation, Transport, and Catabolism. In contrast, genes for Membrane Transport were higher expressed in Porsangerfjorden, although this category also showed high variation within the fjord.

We characterized stations 9 and 10 as “peak-bloom” stations within Balsfjorden, based on the highest recorded *C. leadbeateri* cell densities, whereas stations 7 and 8 are considered as “pre-bloom” because peak cell densities occurred later. Genes of *C. leadbeateri* assigned in the KEGG pathways to Endocytosis, Phagocytosis, Lysosome, and Autophagy (all relevant processes for mixotrophy) were higher expressed in Balsfjorden than in Porsangerfjorden and particularly at the bloom-peak stations 9 and 10 (Fig. 2). Furthermore, KEGG pathway genes characteristic for growth and death processes were higher expressed in Balsfjorden. This indicates that increasing cell numbers and generation of high biomass are facilitated by high expression of genes involved in cell division. The highest expressed genes (KEGG pathway level 3) in Balsfjorden were those coding for the ribosome, indicating enhanced protein biosynthesis and growth, followed by induction of metabolic pathways and biosynthesis of secondary metabolites (fig. S9). The relatively low ambient N- and P-nutrient concentrations at peak bloom stations 9 and 10, compared with pre-bloom stations 7 and 8 within the same fjord, were associated with higher expression levels of primary metabolic and genes involved in translation at the pre-bloom stations and higher levels of mixotrophic genes within the peak bloom.

### Metabolomic integration with metatranscriptomic characterization of microalgal communities

Metabolomic analysis by Fourier transform ion cyclotron resonance mass spectrometry (FT-ICR-MS) detected 7763 features with unique molecular formula assignment. Reconstruction based on UniprotKB-annotated mass differences generated a draft metabolic network encompassing 129,546 edges from 237 assigned mass differences, with all edges having 11,983,272 connections to 1405 UniprotKB entries. A subset of 566 UniprotKB entries was connected to 78 KEGG metabolic pathways, merged into 17 broader classes. The Balsfjorden metabolomes were characterized by KEGG pathways of actively metabolizing cells in conjunction with the metatranscriptomics of a proliferating population.

**Fig. 3.**
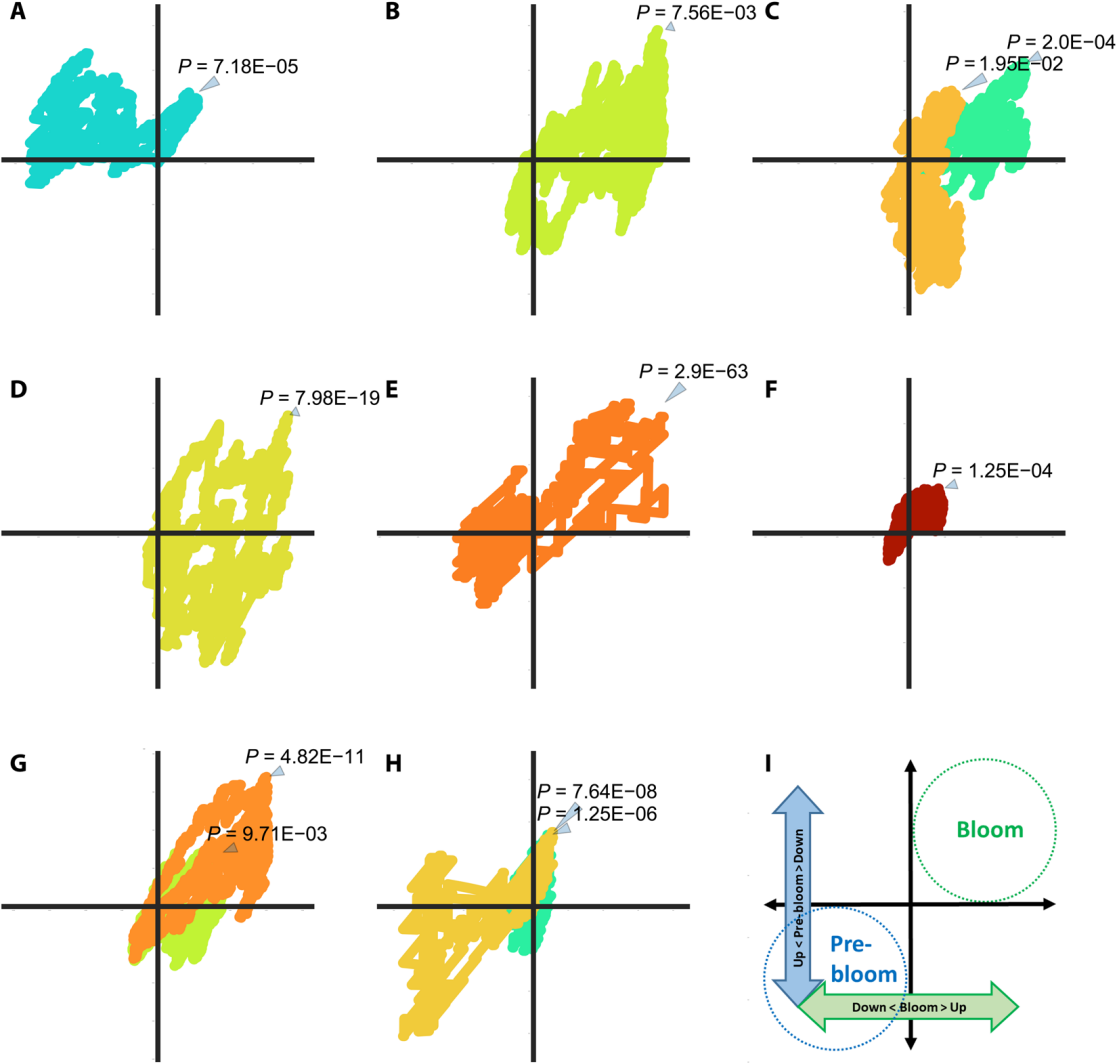
GSVAm walk differences (residuals) between pre-bloom and bloom stations in Balsfjorden. Stations 7 and 8 identified as “pre-bloom” are plotted over the corresponding GSVAm walk of the characterized “peak bloom” St 9 and St 10. Eleven KEGG pathways organized following 8 manually curated pathway categories with GSVAm walks showed deviations toward Quadrant 1 indicating dominant pathways during the bloom (*P* < 0.05; *P* value for maximum deviation from zero). Quadrant 3 indicates negative enrichment amounts of metabolites in the bloom and less negative or even positive enrichment during the pre-bloom period. (**A**) Amino acid metabolism. (**B**) CYP450. (**C**) Carbon metabolism. (**D**) Extracellular matrix. (**E**) Phosphorus metabolism. (**F**) Photosynthesis carbon. (**G**) Secondary metabolism. (**H**) Vitamins. (**I**) Schematic overview.

Meta-metabolomic analyses linked to the underlying gene expression patterns revealed differences among bloom versus pre-bloom stations in Balsfjorden (Fig. 3) and between the respective fjord systems. The gene-based set variation analysis (GSVA) results are displayed from the strip plots of GSVAm walk percentiles of the Balsfjorden and Porsangerfjorden stations (fig. S10). The GSVAm plot shows particularly strong dominance for biosynthetic pathway categories for Secondary metabolites and for Carbon, Energy, Vitamins, and Co-factor metabolism in Balsfjorden. N- and P-metabolic pathways were also relatively enriched in Balsfjorden compared to Porsangerfjorden. Here, it is noteworthy that processes related to Extracellular matrix and Cytochrome p450 (CYP450)–related biodegradation, as well as Signaling pathways, are in line with the HCS cell toxicity results and the presence of LBT-1 from liquid chromatography (LC)–HR-MS/MS (as cited above and shown in fig. S6; fig. S7).

A general overview of residuals of the Balsfjorden bloom at St 9 and St 10, minus the pre-bloom at St 7 and St 8 shown in the GSVAm walks over the corresponding bloom period (Fig. 3), indicates the relative enrichment or unrepresented biosynthetic processes within the fjord. Terpenoid backbone biosynthesis identified with five photosynthesis-related KEGG pathways for photosynthetically derived carbon (C) sources was highly active at bloom St 9 and St 10 (shown in Quadrant 1). The most significant (*P* = 1 × 10^−63^) walks into Quadrant 1 defined by the bloom are found for phosphorus (P) metabolism. Inorganic P concentrations became progressively more potentially growth-limiting from pre-bloom station 7 and 8 to bloom stations 9 and 10 within Balsfjorden (Fig. 1). In contrast, expression of N-metabolism was significantly reduced in all Balsfjorden samples although higher compared to Porsangerfjorden (fig. S11). The second most significant (*P* = 8 × 10^−19^) pathways shown in Quadrant 1 during the *C. leadbeateri* bloom are associated with biosynthesis of polysaccharides that belong to the extracellular matrix, followed by those for secondary metabolites (*P* = 4.8 × 10^−11^), phenylpropanoids in particular (*P* = 4.8 × 10^−11^). Biosynthesis and metabolism of vitamins and coenzymes [CoA biosynthesis/pantothenate (*P* = 7.64 × 10^−8^] and folate biosynthesis (*P* = 1.25 × 10^−6^) were strongly enriched at pre-bloom stations but negatively enriched during the bloom (Quadrant 3). Further enrichments within the bloom encompass metabolic pathways for amino acids (glutamine and glutamate in particular), carbon metabolism of the sugars fructose/mannose via the pentose phosphate pathway (PPP), CYP450, for extracellular biodegradation of aromatics and conjugated pi-bond systems, and macrolide biosynthesis.

**Fig. 4.**
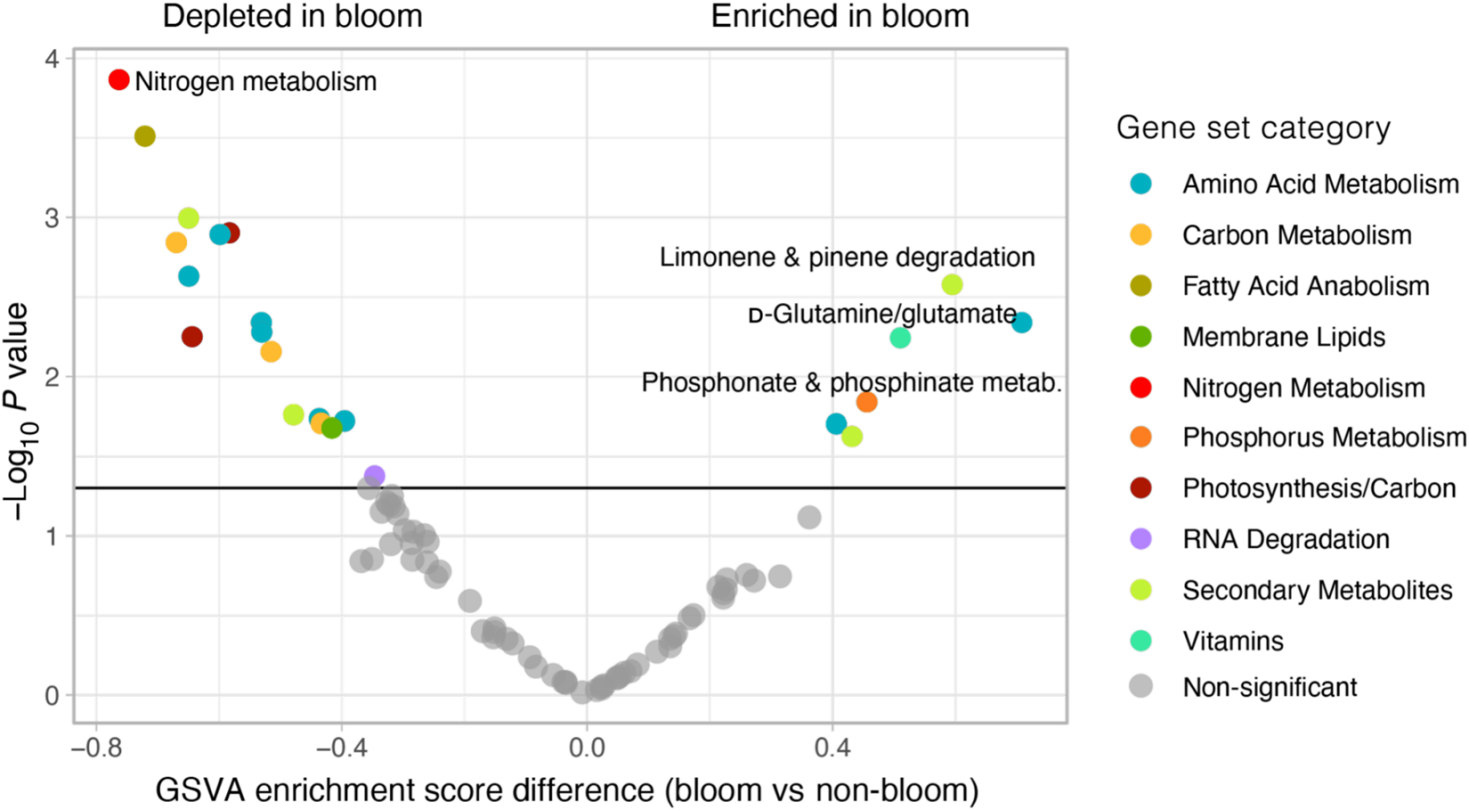
Gene-based GSVA plot of bloom versus pre-bloom stations within Balsfjorden. Negative enrichment score differences indicate a relative reduction in metabolic activity for the respective groups in the bloom, whereas positive values are higher in the bloom (compared to pre-bloom). Gene sets are colored according to corresponding set for metabolomic variation analysis (compare legend on the right). The categories that also show a significant change in the metabolomic analysis are indicated by labels next to the respective colored dot.

The gene-based GSVA within Balsfjorden (Fig. 4) supports our interpretation of enhanced metabolism of amino acids, vitamins, and P-metabolites in the bloom: corresponding gene sets d-glutamine/glutamate (*P* < 0.001), folate biosynthesis (*P* < 0.01), and phosphate and phosphinate metabolism (*P* < 0.05). A lower number of N-metabolites and those associated with photosynthetic pathways and intermediary C-metabolism were identified in samples from bloom stations, in congruence with a lower expression of genes involved in N-metabolism (*P* < 0.001), C-fixation in photosynthetic organisms (*P* < 0.01), and 2-oxocarboxyclic acid pathways (*P* < 0.01), respectively.

### PKS-like genes associated with cytotoxigenicity and putative ichthyotoxicity

A peptide homology search based on the transcriptome of cultured *C. leadbeateri* strain UIO393 revealed 42 potential ketoacyl synthase (KS) domains containing contigs longer than 300 base pairs (bp) and expressed within the bloom study area in northern Norway (fig. S12). All identified KS domains of these sequences were phylogenetically assigned within one of 12 clades of haptophyte PKS genes (fig. S13). The identified KS domains belong to PKS-like multienzymes containing a variety of other domains (fig. S12). No PKS gene contig contained an acyltransferase (AT) domain, but the presence of five AT domains in the transcriptome and metatranscriptome on isolated contigs suggests that they are expressed as independent proteins.

**Fig. 5.**
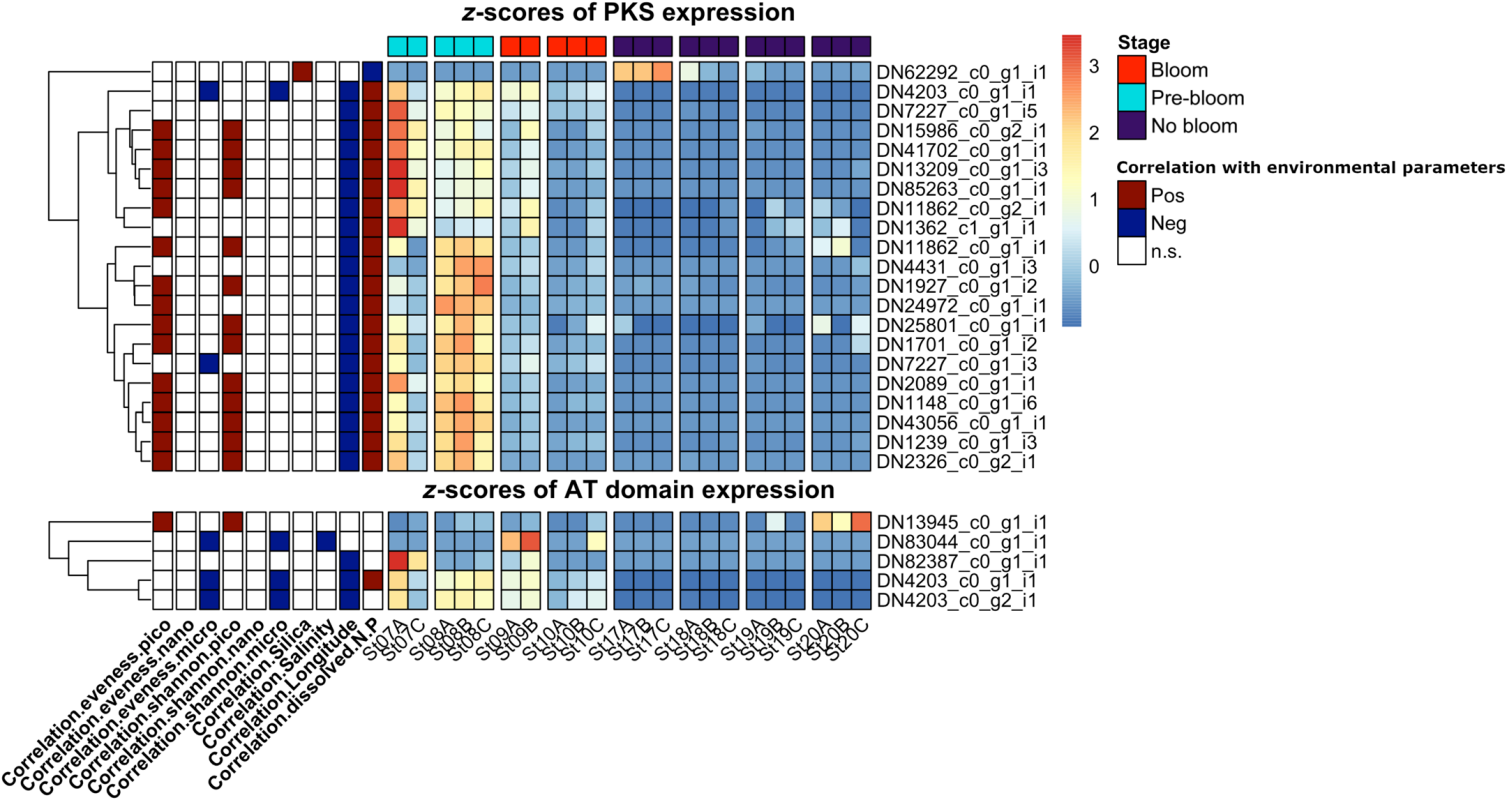
Gene expression (*z*-scores across all samples for each gene) of PKS-like contigs identified in natural phytoplankton communities dominated by *Chrysochromulina* and that correlate with N:P ratios and all identified AT domains. Rows are clustered by similarity within PKS genes (**Top**) and AT genes (**Bottom**). At the top, bloom stage is color coded. On the left, positive or negative correlations of linear models (*P* < 0.05) comparing gene expression with environmental parameters. Shannon diversity and evenness indices were split into operationally defined micro- (>20 μm), nano- (3 to 20 μm), and pico- (0.2 to 3 μm) size-fractions; dissolved silicate (SiO_4_^−^), salinity, longitude, dissolved inorganic N:P ratio (red: positive correlation; blue: negative correlation; white: n.s., not significant).

We identified correlations between the expression of PKS contigs and environmental parameters based on linear regressions correlating the *z*-score of expression across different stations and respective environmental factors (Fig. 5). Twenty-nine of 42 PKS contigs were significantly up-regulated at Balsfjorden stations (correlation with longitude, *P* < 0.05; table S5). Figure 5 shows PKS contigs correlated (*P* < 0.05) with dissolved N:P ratios, of which all but one are higher expressed in Balsfjorden. Most contigs showed highest expression during pre-bloom phase at St 7 and St 8. Of the five identified AT domains, three were higher expressed at Balsfjorden stations (Fig. 5). One PKS contig (DN62292_c0_g1_i1) was higher expressed in Porsangerfjorden and correlates positively with SiO_4_^−^ concentration (*P* < 0.05), an essential nutrient for diatoms. Adding biodiversity data based on metabarcoding (*12*) to the scenario for the Norwegian fjords, we found that diversity and evenness in the picosize cell fraction (0.2 to 3 μm) was reduced with the expression of 16 and 14 genes, respectively (compare table S6 for diversity and evenness data).

Coexpression analysis of the 39 PKS-like genes could not confirm any similar expression patterns for eight putative genes. The remainder formed clusters from one gene to the set limit of 1000 genes, but most genes formed smaller clusters. Seventeen PKS genes with expression patterns correlated with environmental parameters were further analyzed. Most KEGG pathways were represented in clusters of coexpressed genes; an enrichment analysis showed whether pathways were more abundant in a cluster than statistically expected. Three clusters were enriched for “Cell cycle” (*P* < 0.05 or *P* < 0.01), two for “cAMP signaling pathway” (*P* < 0.01), and one each for “Biosynthesis of cofactors” (*P* < 0.05) and “Cysteine and methionine metabolism” (*P* < 0.05; table S7).

## DISCUSSION

The timing, location, and magnitude of ichthyotoxic haptophyte blooms in Norwegian coastal waters (*31*–*33*) have been previously associated with stratified conditions with a low salinity surface layer, increasing solar irradiance, a high N:P ratio, and low inorganic P supply in late spring. The *C. leadbeateri* bloom in 2019 that eventually covered large areas and many fjords in northern Norway was probably initiated in the Lofoten archipelago and Vestfjorden and then progressively spread northward (*12*, *34*) (Fig. 1). On the basis of these general advective processes and spatiotemporal bloom development patterns, St 9 and St 10 within Balsfjorden are representative of “peak bloom.” We consider that conditions at St 7 and St 8 during our sampling period characterize a “pre-bloom” regime because maximum *Chrysochromulina* cell densities and consequent fish mortalities occurred later (https://hi.no/en). Specific abiotic environmental drivers for bloom formation and sustainment are still unclear, but short episodes of heavy rainfall with abundant freshwater runoff and calm weather caused increased stratification in the fjord systems, with a reduced salinity surface layer in the (pre-*Chrysochromulina*) spring bloom period (*12*). Increased freshwater runoff in spring also decisively changed the inorganic nutrient composition and supply and thereby altered growth conditions of the plankton community in fjords affected by the high *Chrysochromulina* cell densities (*12*).

The metatranscriptomic data presented herein indicate that *C. leadbeateri* can plausibly enhance facultative mixotrophy as partial compensation for decreasing inorganic nutrients as bloom cell densities increase. This interpretation is consistent with the haptophyte dominance of a mixotrophic under-ice phytoplankton community, where phagotrophy apparently played a critical role in bloom development in a freshwater-influenced, nutrient-dilute, and low light environment in the Arctic (*35*). In our northern Norwegian ice-free fjord study, cellular processes characteristic of mixotrophy, such as endocytosis, phagocytosis, lysosomal activity, and autophagy, were more prominently expressed in *C. leadbeateri*–dominated populations compared to pre-bloom conditions in Balsfjorden, as well as to non-bloom Porsangerfjorden stations. During the field sampling period, Balsfjorden was potentially N-limited (low DIN) for microalgal growth (Fig. 1), and toward *Chrysochromulina* bloom-dominated St 9 and St 10 ambient inorganic P (DIP) concentrations were also low. Nevertheless, total particulate organic nutrient ratios (POC:PON; POC:POP) did not differ between these stations (fig. S10). Furthermore, nutrient ratios did not deviate much from the classic Redfield ratio (C = 106:N = 16:P = 1) (*36*) for oceanic phytoplankton biomass, i.e., they do not indicate either N or P macronutrient limitation at the cellular level. However, as *C. leadbeateri* achieved dominance in cell numbers and biomass, even at decreasing inorganic macronutrient levels, impending nutrient limitation may have triggered mixotrophic processes to resupply internal reserves and thereby facilitated the bloom. We contend that *C. leadbeateri* enhances mixotrophy as a strategy to compensate limitations of macronutrients such as DIN, DIP, and perhaps vitamins, at the bloom peak, and acquires supplementary fixed DOC and DIC for sustaining the bloom phenotype via this mechanism.

Growth processes expressed by cell proliferation and active metabolism are critical to develop and maintain haptophyte blooms at high cell densities. In Balsfjorden, nearly all key growth-relevant processes from the *C. leadbeateri* event and defined from the metatranscriptomic analysis are reflected by the GSVAm metabolome. In contrast to the metabolomic analysis, the gene-set based GSVA reveals a depletion of macrolide biosynthesis, whereas the metabolomic GSVAm indicates a clear enrichment. Both datasets, however, congruently indicate enrichment of metabolites and genes involved in the biodegradation of aromatic and conjugated pi-bonded compounds in the bloom. Increased C-fixation by photosynthesis was evidenced by enhanced carotenoid, porphyrin and chlorophyll metabolism, and terpenoid backbone synthesis. Additional primary carbon metabolic pathways identified from the metabolome were also expressed in the metatranscriptome. Sugar metabolic pathways, e.g., herein assigned to fructose, mannose, and the PPP for reductive biosynthesis, were overrepresented during the *Chrysochromulina* bloom. These sugar pathway enhancements indicate high metabolic activity associated with cell growth, whereas other C-metabolism processes were rather reduced in both metatranscriptomic and metabolomic expression. Higher expression levels of primary metabolic and translational genes at pre-bloom stations and higher mixotrophic gene expression during the bloom peak suggest a shift to compensate for potential nutrient limitation or starvation, whereas expression of cell cycle genes remains stable. We acknowledge that certain metabolites identified in this study may have been produced by other plankton species found in the 3- to 20-μm size-fraction. Nevertheless, as *C. leadbeateri* was dominant in cell numbers as well as transcriptomic reads at the selected stations, we are confident that general metabolic trends identified here reflect the metabolism of this species.

One early laboratory study has shown enhancement of hemolytic activity and cell biomass of *C. leadbeateri* grown in culture on a polyamine substrate and even suggested that polyamines released from dying or decaying fish during fish-killing events at high stocking densities in confined areas could cause or enhance *Chrysochromulina* blooms in Norwegian coastal waters (*23*). However, the polyamine hypothesis is likely of marginal importance for supporting heterotrophic growth of the virtually monospecific 2019 bloom of *Chrysochromulina* described herein and in the broader regional study (*12*). Polyamines released by phytoplankton blooms typically occur at only nM concentrations in coastal waters (*37*), and although we have no direct measurements of polyamines during the *Chrysochromulina* bloom, the timings, locations, and density of fish kills do not fit this scenario.

Membrane-lytic compounds acting as putative ichthyotoxins in high magnitude blooms are produced by several haptophytes, including close phylogenetic relatives of *C. leadbeateri*, such as *P. parvum* (*38*) and *P. polylepis* (*26*). Previous species interaction studies on *P. parvum* (*15*) and *P. polylepis* (*14*) strains with polyketide biosynthetic capacity (*30*) demonstrated a broad spectrum of membrane lytic activity in cell bioassays and species coculture but only with small-scale cultures applied to experimental cell-based bioassay systems (*28*, *38*) under controlled conditions. Differential expression of lytic toxigenicity of haptophytes, specifically for *P. parvum*, has been shown to depend on nutrient stoichiometry (C:N:P), degree of P and N limitation, and abiotic factors, such as salinity and ambient pH (*29*, *30*, *39*). The biotic mechanisms affecting haptophyte toxigenicity have not been conclusively demonstrated for natural blooms.

Both metaomics datasets herein from a natural ichthyotoxic haptophyte bloom and previous species interaction studies in cocultures support the inference that induction and up-regulation of key secondary metabolic pathways can yield allelochemicals (including “toxins”) associated with competition and cell defense. In the *C. leadbeateri* bloom study reported herein, lytic toxicity in Balsfjorden samples positively correlated with dominance by high *C. leadbeateri* cell densities (>2.0 × 10^7^ cells liter^−1^) whereas no cytotoxicity was recorded from Porsangerfjorden samples with only low *Chrysochromulina* cell densities (<5.0 × 10^3^ cells liter^−1^). Our current results support the hypothesis that such lytic substances, in addition to their ichthyotoxic potency (*40*, *41*), act as allelochemicals to displace competitors, provide chemical defense from predators, and/or to aid in mixotrophic acquisition of immobilized prey (*26*, *28*, *38*). Potential prey items include other microeukaryotes, such as *Cryptophyceae*, *Mediophyceae*, *Dinophyceae*, and *Prymensiophyceae*, or bacteria (*12*, *42*) (compare also to fig. S8, which shows transcripts assigned to five other microeukaryotes).

The lytic mode of action and phylogenetic proximity to the ichthyotoxic species *P. parvum* led to the early assumption [reviewed by (*43*)] that the lytic toxins generated by *Chrysochromulina* species might be structurally related if not identical to polyketide prymnesins (*12*, *14*, *30*). This is, however, not the case for *C. leadbeateri*; recent identification of a previously unknown family of polyketide ichthyotoxins (*17*), dubbed LBTs, from this haptophyte are structurally related to membrane-lytic sterolysins from ichthyotoxic dinoflagellates (*44*). In fact, LBTs share greater structural similarity to karlotoxins (KmTx) produced by *Karlodinium veneficum* [(B. Leadbeater and J. D. Dodge) J. Larsen, 2000] (*40*, *41*) and amphidinols of *Amphidinium* species (*45*, *46*) than to haptophyte prymnesins. Although the notable structural similarity of the sterolysins of *Chrysochromulina* and the dinoflagellates indicates a close phylogenetic relationship of their respective PKS biosynthetic genes, convergent evolution is very unlikely. At this point, however, we cannot define an ancient single- or multiple-lateral PKS gene transfer event. This basic structural similarity between haptophyte and dinoflagellate polyketide sterolysins does not necessarily indicate a shared inheritance of the biosynthetic machinery, especially because *Amphidinium carterae* also uses multidomain PKS (*45*), but, in contrast to *Karlodinium*, its chloroplast is derived from a rhodophyte, rather than from a haptophyte.

Extracellular lytic toxicity has been shown to affect diverse grazers and competitors, but not the producer or conspecifics (*18*, *28*, *30*). The ichthyotoxic dinoflagellate *K. veneficum* is proposed to achieve this immunity via a different membrane sterol composition that prevents interaction with the lytic toxin and thus the membrane-disrupting mode of toxicity (*47*). A similar sterol-dependent pore mechanism to prevent self-toxicity may be used by *C. leadbeateri* but has not been conclusively demonstrated in laboratory settings or from unique sterol composition in haptophytes. The metabolomic data from Balsfjorden, in particular at St 10 with highest *C. leadbeateri* cell densities, do indicate enhanced metabolic activity for production of extracellular matrix components (e.g., mucopolysaccharides), which may be another way to protect *C. leadbeateri* from its own lytic substances. We circumstantially link this together with CYP450-enzyme associated processes, commonly yielding extracellular degradation of such substrate-bearing polymers and extracellular debris.

We confirm the presence of LBT-1 from the *C. leadbeateri* bloom and the association of the high bloom cell densities at stations 9 and 10 in Balsfjorden with high LBT-1 levels. Structural analogs of LBT-1 exhibiting distinct patterns of chlorination and sulfation (*17*) were also detected during the *Chrysochromulina* bloom, but structural identities have not been confirmed. The reasons for the 20-fold higher amount of LBT-1 at St 9 than at St 10, despite 35% higher *C. leadbeateri*–like cell counts at the latter station (table S1) are unclear. This apparent quantitative discrepancy between cell density and LBT-1 concentrations may arise from stochastic spatial variation and artifacts, e.g., in obtaining the cell counts from Niskin bottle samples at the Chl_max_ depth, whereas filtered and dissolved fractions for toxin analyses were derived from large volumes of seawater (500 liters). Although also from the Chl_max_ layer, the pumping depth may not exactly match the discrete depth sampled by Niskin bottles for the cell counts. Alternatively, differences in per cell toxin content could also have contributed to the apparent divergence of cell numbers from LBT-1 amount among stations. Microscale influences by abiotic (temperature, salinity, nutrient status, turbulence) or biotic factors (growth, metabolism, cell life cycle stages, predation, etc.) can also directly or indirectly affect the rate of LBT-1 loss via leakage or excretion and/or net biosynthetic rates.

The polyketide structure of LBT is consistent with PKS biosynthesis. All PKS genes expressed in the metatranscriptome from the *C. leadbeateri*–dominated HAB community were unambiguously assigned to haptophytes, but some were related only to PKS genes found in other toxigenic members of the Prymnesiophyceae (e.g., *P. polylepis* and *P. parvum*). PKS-like contigs found in the transcriptome of *C. leadbeateri* UIO393 (fig. S12) are relatively short multienzymes extending to a maximum length of 3304 amino acid residues and obviously represent only fragments. All described PKS-like contigs lack AT domains. Only five AT domains were detected in the transcriptome, all encoded as individual enzymes with no resemblance to multienzymatic PKS-like structures. We interpret this to indicate that *C. leadbeateri* uses a *trans*-AT PKS system, as proposed in (*48*), to synthesize polyketides, including putative ichthyotoxins such as LBT. The expression of genes associated with cell cycle, cAMP signaling, cofactor-biosynthesis, and cysteine and methionine metabolism is co-correlated with PKS gene expression. Although no functional relationship to toxicity could be deduced here, these genes may correspond to high proliferation (cell cycle), protein biosynthesis (cysteine and methionine metabolism), and signaling (cAMP as second messenger), thereby supports high metabolic activity of *C. leadbeateri* and multiple plankton community interactions.

The ecological impact of toxigenic *Chrysochromulina* on the phytoplankton assemblage at the stations with the highest *C. leadbeateri* cell numbers was evident from the drastically reduced abundance of co-occurring species in the fjord habitat during the bloom. The relatively high expression of PKS genes characteristic of toxigenic Prymnesiophyceae in Balsfjorden further supports our contention that PKS-derived polyketide sterolysins such as LBT analogs were involved in both plankton food web interactions and primarily responsible for the massive fish kills observed during the 2019 *Chrysochromulina* bloom event.

Metatranscriptomic and metabolomic studies on phytoplankton assemblages have yielded valuable insights into cellular and ecophysiological processes (*49*, *50*) and are often the first step in identifying previously unknown genes, pathways, or mechanisms that define roles in bloom dynamics. Our multiomics analysis of a *C. leadbeateri*–dominated bloom event in Balsfjorden, involving coexpression analyses of candidate PKS genes and metabolic functions linked to mixotrophy, provides insights into how cellular processes and environmental factors are co-correlated with bloom phases. The environmental factors inducing *Chrysochromulina* toxigenicity in the field are still not well understood and reliable induction of toxigenicity with little knowledge of the causative metabolites is not readily accessible from laboratory culture studies. Further laboratory studies are essential to confirm correlations as causalities, e.g., the direct functional role of LBT in the cellular lysis of eukaryotes and prokaryotes and subsequent mixotrophic nutrient uptake. A caveat of our study is that, in contrast to transcriptomic reads, metabolites and particulate carbon/chlorophyll biomass cannot be uniquely assigned to *C. leadbeateri* but may be produced by other species. Stable isotope analysis, as done in (*51*), would additionally allow us to assign N and C uptake to autotrophic or heterotrophic processes. Unfortunately, this analysis was not possible to include in the advance cruise planning or apply retroactively due to the unforeseen encounter with the massive *Chrysochromulina* bloom during the oceanographic expedition in northern Norway.

Even when abiotic factors provide an optimal environment for *Chrysochromulina* bloom initiation, how HABs of ichthyotoxic haptophytes can achieve such high cell densities and persisting over several weeks remains elusive. At this stage, we propose a cell–based conceptual biotic model derived from the *C. leadbeateri* case study presented herein, which is consistent with our metaomics and toxigenicity analysis. Under this scenario, an initial pre-bloom *Chrysochromulina* population in a diverse plankton assemblage invokes life-style nutritional transition mechanisms, leading to enhanced mixotrophy and induction of cell toxigenicity as inorganic nutrient competition and grazing pressure intensifies. Increased production of cytolytic allelochemicals such as LBT, and mucopolysaccharide at the cell surface, coupled with extracellular digestive enzyme activity, reduces grazing. Cytolytic effects on membrane fluidity also facilitate mixotrophic processes associated with prey capture, phagocytosis, and metabolism and extracellular degradation and recycling of particulates and dissolved organics. Mixotrophic acquisition of organic nutrients from grazers, competitors, and neutral prey, including dissolved and particulate matter from allelochemically immobilized and lysed cells, yields a net growth advantage for bloom formation, dominance, and maintenance. Coupled modeling studies incorporating mixotrophy [e.g., (*52*)] are crucial for interpreting the dynamics of HABs and their associated fish kills, with implications for both ecosystem health and commercial fisheries and aquaculture. Developing a comprehensive understanding of these factors will also assist in mitigating the adverse effects of toxic haptophyte blooms on marine ecosystems.

## MATERIALS AND METHODS

### Integrated study design and approach

The field component of this integrated study comprised a comparison of two fjord systems during a major fish-killing bloom event in northern Norwegian waters (Fig. 1). We conducted oceanographic measurements during the *RV Heincke* HE533 expedition (*53*) between 25 May and 30 May 2019 at all stations during the entire research cruise, and collected plankton and seawater samples at selected stations within Balsfjorden and Porsangerfjorden. We analyzed oceanographic data onboard for initial comparisons of the prevailing environmental parameters within the respective fjord systems. Preliminary microscopic screening of plankton samples identified stations in Balsfjorden as peak bloom locations. As comparison with a non-bloom environment, we chose Porsangerfjorden, a structurally similar fjord system further north, but within which no *Chrysochromulina* bloom or other HAB event occurred during the cruise period or apparently thereafter.

We applied a multiomics approach to plankton components collected during the *Chrysochromulina* bloom period to identify key pathways and metabolites, including potential ichthyotoxins. Metatranscriptomic profiles for the dominant *Chrysochromulina* genotypes served to identify underlying gene expression of these taxa. We identified putative toxin-producing genes based on homology with PKS of the closely related ichthyotoxic *P. parvum*. Last, we integrated metatranscriptomic and metabolomic data between fjord systems and then correlated with environmental and oceanographic parameters to generate a plausible biotic model for *Chrysochromulina* bloom development. The objective was to achieve a better holistic understanding of biological causes and metabolic drivers for this unprecedented harmful haptophyte bloom.

### Oceanographic data and field sample collection and archiving

Oceanographic data on abiotic factors, and seawater samples containing plankton, were collected from depths between 3 and 40 m with a CTD rosette sampler (SBE911+ CTD, Sea-Bird Electronics, Bellevue, USA). The CTD was equipped with duplicate sensors for temperature (SBE3plus) and conductivity (SBE4) as well as an oxygen sensor (SBE43). Additional sensors, including a C-Star transmissometer and ECO-AFL fluorometer (Sea-Bird Electronics, Bellevue, USA, former WETLabs), and an altimeter (Teledyne Benthos PSA-916, North Falmouth, USA) were mounted to the CTD. Environmental data on abiotic oceanographic parameters, in vivo chlorophyll, and inorganic macronutrients (N, P, and Si) are available in the HE533 cruise Data Processing Report (*54*) and are archived in the Pangea database (https://pangaea.de/).

Seawater samples (15 liters) in triplicate were collected with Niskin bottles with the rosette sampler from four key stations within Balsfjorden (St 07 to St 10) and Porsangerfjorden (St 17 to St 20) from three discrete depths (surface, ~3 m; Chl_max_, deep water, ~40 m). Samples for extracted chl a measurement were filtered onboard through a 0.7-μm pore size, glass fiber filters (Whatman GF/F) under low vacuum. Filters were frozen immediately at −80°C until chlorophyll extraction in 90% acetone. Chl a concentrations were determined according to an EPA standard method (EPA445.0, Arar & Collins, 1997), calculated from fluorescence measured with a precalibrated Turner Designs TD 700 fluorometer (Turner Design Inc., San Jose, CA, USA).

Seawater samples (50 ml) collected with Niskin bottles from the chlorophyll maximum layer were fixed with Lugol’s iodine solution (1% final concentration) for plankton identification and cell counts. After sedimentation for 24 hours in a 50-ml Falcon tube, the top 45 ml was aspirated away. A subsample (0.1 ml) of the 10X concentrate was transferred to a Palmer-Maloney counting chamber (PhycoTech Inc., St. Joseph, MI, USA) for onboard analysis under a light microscope (Zeiss AxioPlus, Jena, Germany). *C. leadbeateri* cells were reliably identified by phase-contrast microscopy (400X magnification) based on their characteristic cell size, round shape, the presence of two chloroplasts, two long flagella, and a coiled haptonema slightly longer than the flagella (*12*).

Filtered plankton components were processed and archived for multiomics as described in detail for a previous field study in northern Norwegian fjords (*12*). Filters for the RNA metatranscriptomic analysis were immediately placed in 1 ml of TRIzol (Thermo Fisher Scientific, Darmstadt, Germany) for maintaining RNA integrity while disrupting cells and dissolving cell components, and then stored −80°C. Sample filters for metabolomics and putative cytotoxicity analysis immediately frozen and stored at −80°C prior to extraction.

### Extraction, sequencing, and RNA transcriptome processing

Cells from filters were lysed with a bead beater (MagNA Lyser, Roche, Basel, Switzerland) using glass beads. RNA was further isolated according to a TRI Reagent–based method supported by linear acrylamide as described in (*55*). RNA quality was examined with a LabChip (PerkinElmer, Waltham, USA).

A reference transcriptome of *C. leadbeateri* strain UIO393 (Norwegian Culture Collection of Algae, NORCCA) was obtained from separate cultures grown at 13°C in modified IMR1/2 and L1 culture media at salinity 34 and IMR1/2 at salinity 25, respectively. RNA was extracted with the RNAqueous Total RNA Isolation Kit (Thermo Fisher Scientific, Waltham, MA, USA) with DNase I digest and cleaned with the RNeasy MinElute Cleanup Kit (Qiagen, Hilden, Germany). RNA libraries were generated with the Illumina Stranded mRNA Prep kit (Illumina, San Diego, CA, USA), according to the manufacturer’s instructions, using IDT-Illumina RNA UD Indexes Set A and B indices. Libraries were sequenced with a NovaSeq SP (250-bp paired-end reads) at the Norwegian Sequencing Centre, University of Oslo for the reference transcriptomes or in a NextSeq flow cell (150-bp paired-end reads) at Alfred Wegener Institute, Bremerhaven for the metatranscriptomes. Sequences were trimmed by cutadapt v1.8.3 and assembled with Trinity v2.9.1. Open reading frames of >100 amino acids were predicted with a getorf script in six frames (EMBOSS v6.6.0) and Transdecoder (v5.5.0.). Transcriptomes were annotated using a trinotate pipeline, including transdecoder, the SwissProt database, and UniProt [uniprot.org (*56*)] and KEGG (https://genome.jp/kegg/). Metatranscriptome reads were mapped to transcriptomes in CLC Genomics Workbench (version 20.0).

### High-content screening

We performed HCS for bioactivity of our samples against human sarcoma cell line U-2 OS with the cell painting assay according to (*57*) (for full methodological details, see text S1). Briefly, 979 features of morphological (STAR and Shape) and texture (SER) characteristics were recorded in triplicate of each sample on an Operetta High Content Imaging System (PerkinElmer, Hamburg, Germany) equipped with software Columbus (2.9.1532). The Grubbs outlier test was used for determining outlier features in negative controls. Features were tested for significant bioactivity differences between the plankton cell samples collected pre-bloom, at peak bloom, and at non-bloom stations. Artifacts were controlled for by applying a Kruskal-Wallis test with Benjamini-Hochberg controls for false discovery rate corrected for multiple testing (*P* < 0.05). Statistical analysis was done in R studio version 2021.09.0. We interpreted effects on the cell line U-2 OS as a proxy for cytotoxicity in the respective fjord plankton samples.

### Metabolomics analysis by FT-ICR-MS

Freeze-dried filtered field plankton were extracted by homogenization in ultrapure water:MeOH (4:1). After further processing, methanolic extracts were analyzed by FT-ICR MS (Bruker Daltonics, Bremen, Germany) equipped with electrospray ionization in negative and positive ion mode (for full methodological details, see text S2). Three-hundred scans were acquired at 4 MegaWord (MW) time domain length. Representative spectra were subjected to mass difference network (MDiN)–based molecular formula assignment (*58*). Resulting high-confidence molecular formulas were recalibrated using mass difference kernels (*59*) on all remaining spectra. The resulting mean *m/z* values per feature were subjected to MDiN-based molecular formula assignment and then to further bioinformatics analyses for confirmation.

### Constructing MDiNs

MDiNs were constructed as a model for the global biochemical network that governs the meta-metabolomes of the samples. An MDiN is a graph *G*(*N*,*E*) with nodes *N* being the metabolic features detected by FT-ICR-MS and edges *E*(*i*,*j*) being assigned if

m⟦e⟧=m⟦j⟧−m⟦i⟧∣m⟦e⟧∈K (1)

where m[.] is the exact molecular mass and *K* is a list of *k* mass differences (MDs). The transformation was performed using the geometric mean of source and target node of edge r to compute *U*(*r*). One master MDiN was built for the entire sample set *X* that yields a master edge dataset *U* recovering the individual MDiNs for each sample *S*. The matrix *U* is the enzyme/pathway annotated projection of *X*, subject to statistics that leverage the MDiN.

The MDiN annotations shed light on metabolic pathway utilization from the meta-metabolomic perspective, in this case annotated with reference to archived transcriptomic data. The retroRules database (*60*), which covers >400,000 chemistry-aware reaction rules, was mined from MetaNetX (*61*, *62*), and cross-linked to RheaDB, Metacyc, and UniProt amino acid sequences. Herein, all rules at diameter 6 to mine reaction substrates and products from various databases were used to compute the mass difference for each substrate-product pair, resulting in an enzyme/gene/UniprotID-annotated list of mass differences for MDiN reconstruction. Detailed script information can be found under DOI:https://doi.org/10.5281/zenodo.14283884.

### Gene set variation analysis on metabolites and genes

The GSVAm (*63*) was performed on gene sets of interest with the variability of transcriptomic data at hand, based on KEGG annotations and transcripts per million (TPM) values. The gene sets compared in this analysis include the pre-bloom stations (St 7 and St 8) and the bloom stations of Balsfjorden. The analysis was conducted in R [version 4.2.3 (*64*)] with the R package GSVA (*63*). Herein, the gene sets were defined by mapping the KEGG pathway annotations from metatranscriptomics workflow to the UniprotID-annotated MDiNs that span the dataset *U*. The dataset *U* represents an analog to reverse transcription quantitative polymerase chain reaction data that is directly amenable to techniques such as gene set enrichment analysis (GSEA) or gene set variation analysis given a number of adaptations.

Two adaptations to the computation of Kolmogorov-Smirnov (KS) walks and their maximum value (the enrichment score ES) were implemented to accommodate biases and the fact that one MD in K might be associated with more than one enzyme belonging to the same gene set $\gamma_{l}$.

The final formulation for the KS-like random walk reads as

$$\begin{array}{c} {Ε}_{\mathrm{sl}}\left(w\right)=\frac{\sum_{r=1}^{w}{|\;U_{\mathrm{rs}}\;|}^{\tau }C\left(g_{ra}\in \gamma_{l}\right)}{\sum_{r=1}^{R}{|\;U_{\mathrm{rs}}\;|}^{\tau }C\left(g_{ra}\in \gamma_{l}\right)}-\frac{\sum_{r=1}^{w}{|\;U_{\mathrm{rs}}\;|}^{\tau }C\left(g_{ra}\in \gamma_{l}\right)}{\sum_{r=1}^{R}{|\;U_{\mathrm{rs}}\;|}^{\tau }C\left(g_{ra}\in \gamma_{l}\right)} \end{array}$$ (2)

with $C\left(g_{ra}\in \gamma_{l}\right)$ being the count vector of how often feature *r* received gene set annotation $\gamma_{l}$.

We performed a GSVA based on KEGG annotations and TPM values to facilitate comparison and integration of the metatranscriptome with the metabolomic data. The gene sets compared in this analysis include data from both bloom and non-bloom Balsfjorden stations. The analysis was conducted in R (version 4.2.3, 2023) with the R package “GSVA” (*63*). Detailed script information can be found under DOI:10.5281/zenondo.14283885.

### Identification of the putative ichthyotoxin LBT-1

Targeted detection of LBT-1 was performed for the filtered particulate organic fraction (Whatman GF/F, 0.7-μm nominal pore size, Whatman, UK) and dissolved organic fraction by solid-phase extraction (SPE) Bondesil-ENV, Agilent, USA) of filtered seawater (for full methodological details, see text S3). LBT-1 was detected and confirmed by two alternative mass spectrometry platforms—LC coupled to time-of-flight mass spectrometry (6545 QTOF-MS, Agilent Technologies, Santa Clara, CA, USA) and LC coupled to a high-resolution mass spectrometer (Q Exactive Plus, Thermo Fisher Scientific, Germany) (for full methodological details, see text S2.3). The spectra were compared to LBT-1 extracted from laboratory cultures of *C. leadbeateri* as described by Wang *et al.* (*17*) (fig. S5). No analytical reference standard is available; LBT-1 quantitation is therefore reported herein only as relative concentrations found in the SPE extracts and extracts of the particulate fractions at the respective stations.

### PKS gene identification

A KS (β-ketosynthase) homology search and phylogeny was performed to identify putative polyketide toxin-promoting genes. A peptide homology search for contigs containing PKS or fatty acid synthase (FAS) sequences was conducted on the *C. leadbeateri* transcriptome with HMMER (*65*), *e* value cutoff ≤ 1 × 10^−10^. Manual filtering for contigs > 300 bp and containing ≥1 KS domain was done using the NCBI batch cd search. Sequences were analyzed in Geneious Prime (version 2021.1.1), aligned with the MAFFT algorithm (*66*); a maximum likelihood phylogenetic tree was built using RaxML (*67*) with 100 bootstrap replications. The sequences were aligned with reference groups containing FAS and PKS of haptophyte species *P. parvum* (*30*). Phylogenetic trees were visualized using iTOL (*68*). Domain compositions of predicted PKS-like sequences containing KS domains were annotated by an InterPro scan using Gene3D, Panther, Pfam, SMART, and Superfamily member databases.

### Statistical analysis of metatranscriptome and PKS coexpression

Statistical analyses and visualization of metatransciptomic data were performed in R 4.0.5 (*64*), using the packages ggplot2 (*69*) and pheatmap (*70*). Metatranscriptomic count data were normalized as TPM^0.25^, considering read length and relative abundance of species at each sampled location. Reference transcriptomes for other species were retrieved from the MMETSP project [https://ebi.ac.uk/ena/browser/view/PRJNA248394 and (*71*)]. To achieve normal distribution for statistical tests, read data were either visualized as TPM^0.25^ or *z*-scores were calculated for better comparison. The gene expression values of identified PKS contigs were correlated with environmental parameters based on linear regressions established with the base R command lm(y ~ x). A *P* value of < 0.05 was considered statistically significant. Diversity and evenness data were calculated from 18*S* ribosomal RNA gene metabarcoding using the R Package PhyloSeq (*72*) as in (*12*).

Coexpression of putative PKS genes with similar expression patterns was identified by R packages genefilter (*73*) and Bioconductor (*74*), using “genefinder” with the settings method = “euc,” scale = “none,” cutoff = 1. An enrichment analysis invoked “fold enrichment,” calculated as $\frac{x}{k}/\frac{m}{N}$ , *x* is the number of genes in the selected pathway in the cluster, *k* is the number of genes in the cluster in total, *m* is the total number of genes in the selected pathway in the whole dataset, and *N* is the total number of annotated genes in the pathway.

## Supplementary Materials

### The PDF file includes:

Supplementary Text S1 to S3 Figs. S1 to S13 Legends for tables S1 and S5 Tables S2 to S4, S6 and S7
