# Supplementary material for: High-resolution multiomics links nutrients and mixotrophy to toxicity in a harmful bloom of the haptophyte Chrysochromulina leadbeateri
Source: Sci Adv. 2025 Jun 25;11(26):eadv3390. doi: 10.1126/sciadv.adv3390 (PMC12190008; doi:10.1126/sciadv.adv3390)
Supplement: Supplementary file 1 — Supplementary Text S1 to S3 Figs. S1 to S13 Legends for tables S1 and S5 Tables S2 to S4, S6 and S7 [file sciadv.adv3390_sm.pdf]

Science Advances
AAAS

Supplementary Materials for

High-resolution multiomics links nutrients and mixotrophy to toxicity in a harmful bloom of the haptophyte *Chrysochromulina leadbeateri*

Antonia Otte *et al.*

Corresponding author: Uwe John, uwe.john@awi.de

Sci. Adv. **11**, eadv3390 (2025)
DOI: 10.1126/sciadv.adv3390

The PDF file includes:

Supplementary Text S1 to S3
Figs. S1 to S13
Legends for tables S1 and S5
Tables S2 to S4, S6 and S7

Other Supplementary Material for this manuscript includes the following:

Tables S1 and S5

Supplementary text S1

Methodology: High Content Screening (HCS)

Filtered plankton (mostly of *Chrysochromulina*) cell extracts were subjected to HCS in triplicate with controls to differentiate effects on actin, cytoplasm, endoplasmic reticulum (ER), mitochondria, nucleoli, and the nucleus of human osteosarcoma U-2 OS cells. Samples were randomly positioned among the three replicates, but in the same orientation on both plates for the staining pair. DMSO-treated U-2 OS cells served as negative controls. Images were acquired on a PerkinElmer Operetta® High Content Imaging System, with Perkin Elmer software Columbus (2.9.1532) for feature extraction. In total, 979 features were recorded — comprising the mean value of morphological characteristics (STAR and Shape) and texture (SER), and where possible the standard deviations. The Scrubbs outlier test was applied to determine outlier features for negative controls; if there were more than 30 outlier features, the control sample was removed. Remaining negative control samples were subjected to z-score transformation for all samples to allow for plate comparisons. Finally, individual replicate data were retained and then combined into one data set, containing all features for both negative controls and samples. Highly co-correlated features were avoided by data cleaning (cut-off set to 0.8; 708 features). Statistical analysis was conducted in R studio version 2021.09.0. Features were tested for significant character differences between the bloom-dominated and non-bloom fjords by a Kruskal-Wallis test with Benjamini-Hochberg (BH) controls for False Discovery Rate (FDR) corrected for multiple testing (p value<0.05).

Supplementary text S2

Methodology: Metabolomic analysis of plankton cell extracts by FT-ICR mass spectrometry

The freeze-dried plankton on 3 µm-pore size polycarbonate filters (Millipore, Darmstadt, Germany) stored at -80 °C were cut into 2 mm slices and transferred into 8 mL MilliQ H_2O and 2 mL MeOH (LC-MS grade) in 15 mL Eppendorf vials. Filter flakes were macerated with a homogenizer (Ultra-Turrax T25, IKA, Staufen, Germany) at maximum power for 1 min. Macerates were centrifuged at 3,700 x *g* for 15 min and then supernatants were transferred to 15 mL reaction vials and vacuum dried. Dried samples were reconstituted in 2 mL ultrapure H_2O and ultra-sonicated in a bath sonicator for 15 min. Ethyl acetate was added twice (2 mL) for extraction to H_2O-reconstituted samples in 10 mL glass vials, then vortexed for 1 min and placed in an ice bath for phase separation for 1h. Aqueous phases were transferred to new 10 mL glass vials and extracted twice against BuOH (LC-MS grade), as described above for ethyl acetate. BuOH phases (4 mL altogether) were collected and extracted against another 4 mL MilliQ H_2O to remove traces of salt. The final BuOH phases were vacuum-dried, reconstituted in 1 mL MeOH, aliquoted into 96-well plates (200 µl per aliquot) and vacuum dried for storage until further analysis.

Vacuum-dried samples were reconstituted in 1 mL MeOH for direct injection analysis by Fourier transform ion cyclotron resonance mass spectrometry (FT-ICR MS) (Bruker Daltonics, Bremen, Germany). Reconstituted samples were injected from 96 well plates using a PAL sampling robot at a flow rate of 2 µL min^{-1}. Samples were ionized in both negative and positive mode by electrospray ionization (ESI); 300 scans were acquired at 4 MegaWord (MW) time domain length.

Mass spectra were calibrated against solvent impurities, and provisional molecular formulas were assigned using isotopic fine structure where possible. Representative spectra were subjected to mass difference network (MDiN)-based molecular formula assignment (*52*). Resulting high-confidence molecular formulas were re-calibrated by using mass difference kernels (*53*) on all remaining spectra. Spectra were cleaned from artifacts and aligned into a matrix within a 0.5 ppm mass window with an in-house pipeline. The resulting mean *m/z* values per feature were subjected to final MDiN-based molecular formula assignment.

Supplementary text S3

Methodology: Harvest, detection and relative quantification of leadbeaterin-1 from the *Chrysochromulina* bloom

Field and culture sample collection and processing for mass spectrometry

Seawater was pumped through a water hose attached to a PTFE membrane pump (Steinle, Germany) from approximately 12 m depth near the chlorophyll maximum layer from the bloom-dominated and non-bloom fjord stations. Pumped seawater was retained in a 600 L intermediate bulk container. The seawater was subsequently separated into a particulate organic fraction retained on glass fiber filters (142 mm, 0.7 µm nominal pore-size, GF/F, Whatman, UK), and a dissolved organic fraction by pumping (PTFE-membrane pumps, Fink, Germany) the filtrate through solid phase extraction (SPE)-cartridges (4 x 20 cm; Bondesil-ENV, 125 µm particle-size, Agilent, USA) packed with poly-styrene-divinylbenzene (PSDVB) resin for adsorption of organics. The organic-loaded resin was washed with two column volumes of ultrapure H_2O and frozen at -20°C until further analyses. The particulate organic fraction samples on filters were also frozen at -20°C until further processing. The SPE-cartridges were eluted with 500 mL MeOH (LC-MS grade), then vacuum-evaporated until almost dry and redissolved in 20 mL MeOH (LC-MS grade). The GF/F filters or particulate organic analysis were extracted three times with 150 mL MeOH (LC-MS grade). Extracts were pooled, evaporated until almost dry under vacuum and redissolved in 20 mL MeOH (LC-MS grade).

For characterization and relative quantification of potential ichthyotoxins, mass spectrometric data from the environmental samples were compared with data generated from a highly cytotoxic fraction obtained from *C. leadbeateri* strains UIO 394 and UIO 035 from NORCCA. The *C. leadbeateri* strains were cultured in 10 L under controlled conditions and harvested as cited herein (*17*).

Detection and relative quantification of the putative ichthyotoxin LBT-1

Leadbeaterin (LBT) analogs in the dissolved and particulate organic fractions were detected and confirmed by liquid chromatography (LC) coupled to two independent high resolution mass spectrometry (HRMS) platforms (Table S2, Table S3):

1) A UHPLC system (Infinity 1290) coupled to a time-of-flight mass spectrometer (6545 QTOF-MS) , Agilent Technologies, Santa Clara, CA, USA. Separation was achieved on a 150 × 2.1 mm, 1.9 µm, Poroshell 120 phenyl hexyl column (Agilent Technologies, Santa Clara, CA, USA), held at 40 °C. MS detection was performed with a dual jet stream electrospray ion source (ESI), at a drying gas temperature of 250 °C in positive mode and 325 °C in negative mode, a gas flow of 8 L min^{-1}, a sheath gas temperature and a flow

rate at 300 °C and 12 L min^{-1}, respectively. Capillary voltage was set to 4000 V and nozzle voltage to 500 V in both positive and negative modes. MS spectra were recorded as centroid data, scan range *m*/*z* of 100−1700; auto MS/HRMS fragmentation was performed at three collision energies (10, 20, and 40 eV), on the three most intense precursor-ions per cycle. The acquisition rate was 10 spectra s^{-1}. Data were handled with Agilent MassHunter Qualitative Analysis software (Agilent Technologies, Santa Clara, CA, USA). Tributylamine (1 µM); Sigma–Aldrich, Saint Louis, USA) and Hexakis (2,2,3,3-tetrafluoropropoxy)phosphazene (10 µM; Apollo Scientific, Cheshire, UK) in 70% MeOH were infused as lock mass compounds in a second sprayer using an extra LC pump at 15 µL min^{-1} equipped with a 1:100 splitter. The mass to charge ratio (*m*/*z*) of the $[M + H]^+$ ion (*m*/*z* 186.2216 and 922.0098, respectively), or the *m*/*z* of $[M + HCO_2]^-$ of the phosphazene (*m*/*z* 966.0007) were employed for internal mass calibration.

2) A UPLC system (Vanquish) coupled to a high-resolution mass spectrometer (Q-Exactive Plus), Thermo Fisher, Bremen, Germany. Separation was achieved on a phenyl hexyl column (100 × 2 mm, 1.7 µm, Waters, USA) equipped with a guard-column, under the following settings: Solvent A = 0.1% formic acid in ultrapure H_2O, solvent B = 0.1% formic acid in acetonitrile; 0 min: B = 2%, 4 min: B = 2%, 17 min: B = 99%, 20 min: B = 99%, 21 min: B = 2% with a flow rate of 0.45 mL min^{-1}. The effluent of the first 1.5 min was diverted to waste to limit salt deposits. The column oven was set to 40 °C. The mass spectrometer was calibrated using Positive Ion Calibration Solution (Pierce, Thermo Fisher Scientific); all MS measurements were made in positive ion mode. Data were analyzed in data independent mode using an inclusion list (consisting of the accurate masses for $C_{67}H_{128}O_{27}Cl$ and $C_{67}H_{125}O_{27}Cl_2$) with a full-scan resolution of 140,000 (*m/z* 200) followed by MS2 experiments at RES = 35,000 (normalized stepwise collision energy of 25, 35, 45; automatic gain control target of 2×10^5; and 50 ms maximum injection time).

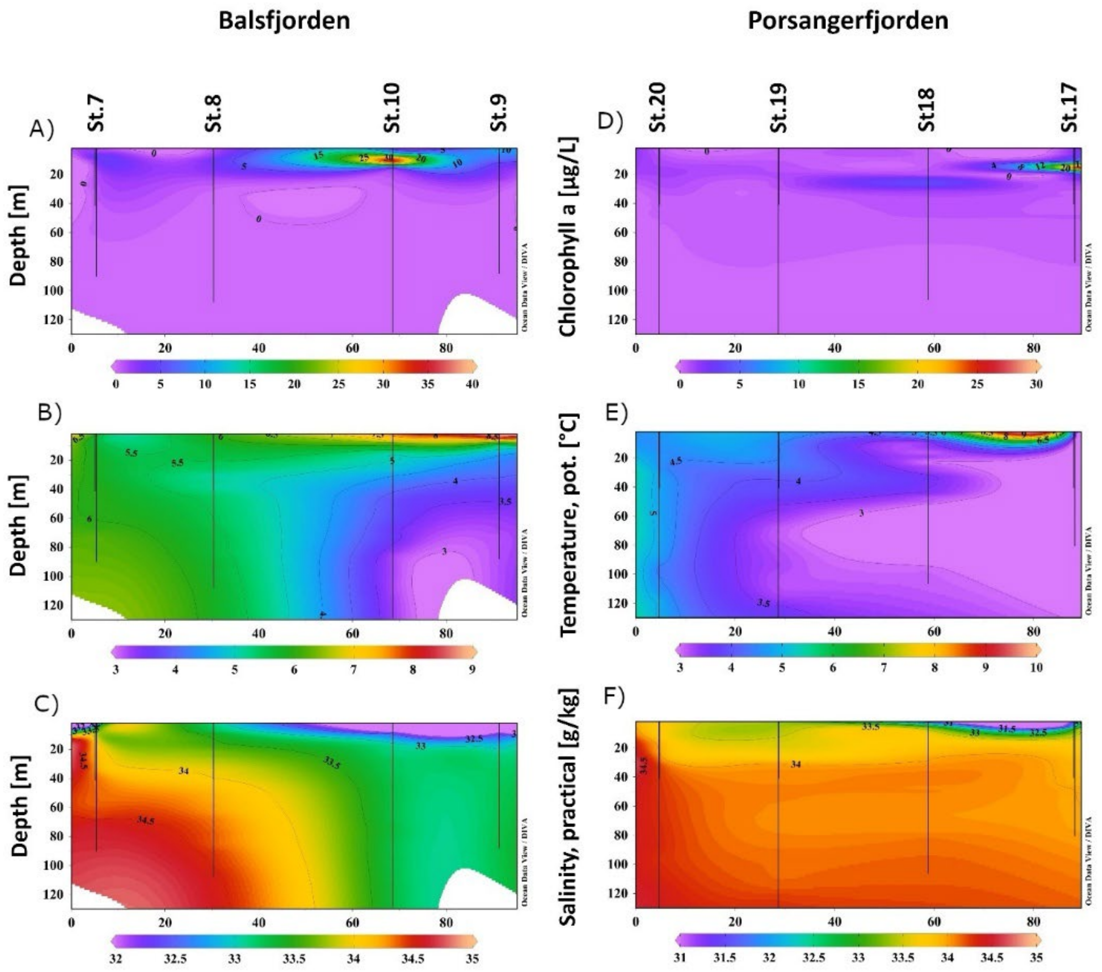

Fig. S1.

Vertical profiles of transects along Balsfjorden (A, B, C) and Porsangerfjorden (D, E, F) displaying chlorophyll a [µg L^{-1}] (A, D) derived from *in situ* chlorophyll fluorescence, potential temperature [°C] (B, E), and practical salinity [g kg^{-1}] (C, F).

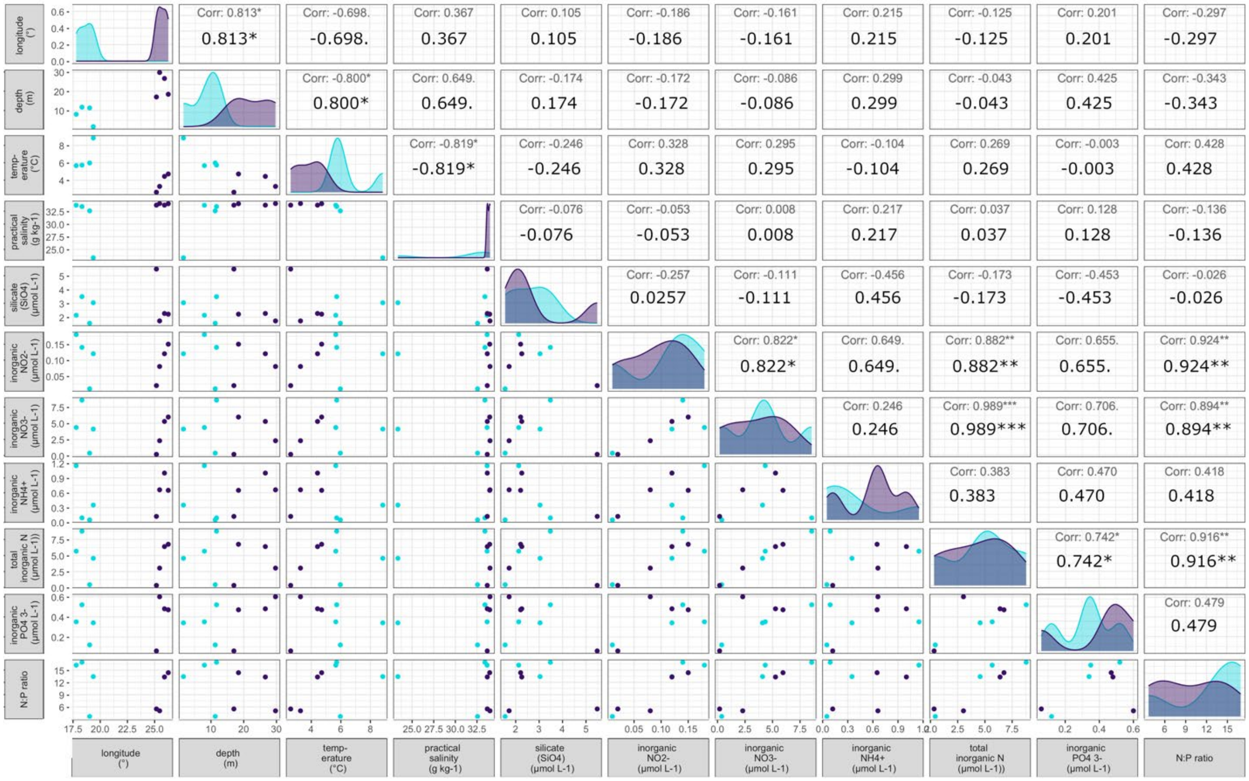

Fig. S2. Correlated environmental parameters in Balsfjorden and Porsangerfjorden.

Pairwise correlation matrix for environmental parameters at the chlorophyll maximum layer: longitude (°), depth (m), temperature (°C), practical salinity (g kg^{-1}), silicate (SiO_4) (µmol L^{-1}), inorganic nitrogen (NO_2^-, NO_3^-, and NH_4^+) (µmol L^{-1}), total inorganic N (µmol L^{-1})), inorganic PO_4^{3-} (µmol L^{-1}), and N:P ratios. Upper triangle: Pearson correlation coefficient; p values marked with asterisks. Significance levels: . : 0.05 - 0.10; *: 0.01 - 0.05; **: 0.001 - 0.01; ***: 0 - 0.001; Diagonal: distribution of values in Balsfjorden (light blue) and Porsangerfjorden (purple); Lower triangle: correlation plots with data from each station, color-coding for fjord as above (for absolute values see Table S1).

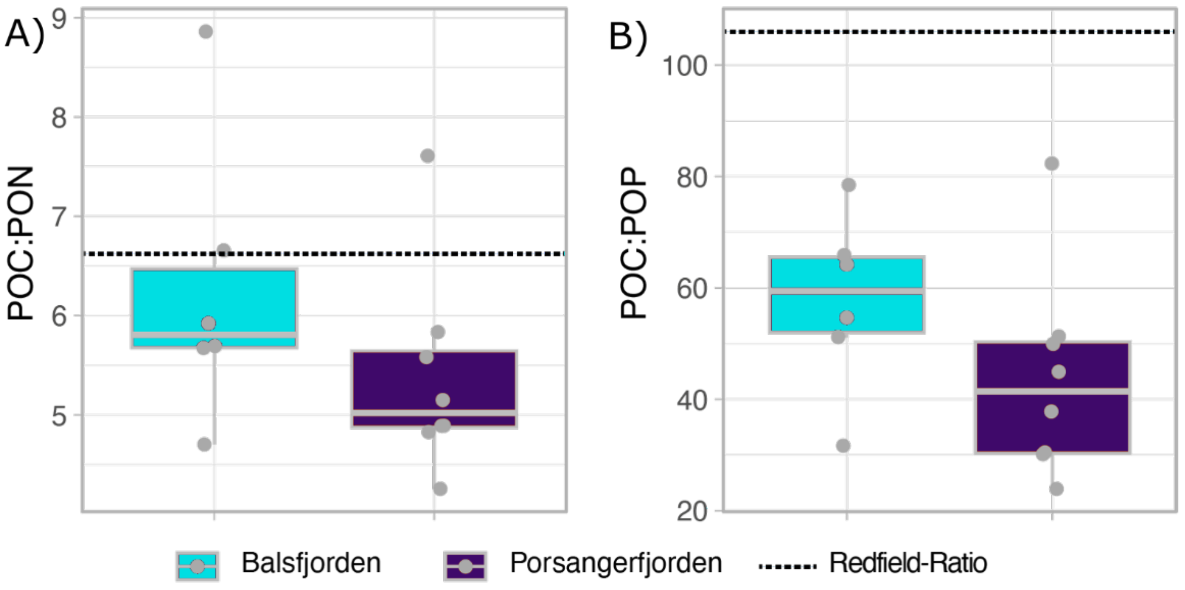

Fig. S3.

Total particulate organic nutrient ratios (POC:PON (A)and POC:POP) (B) determined from Balsfjorden and Porsangerfjorden stations. The dotted line indicates the Redfield-Ratio for the respective nutrient ratio plots.

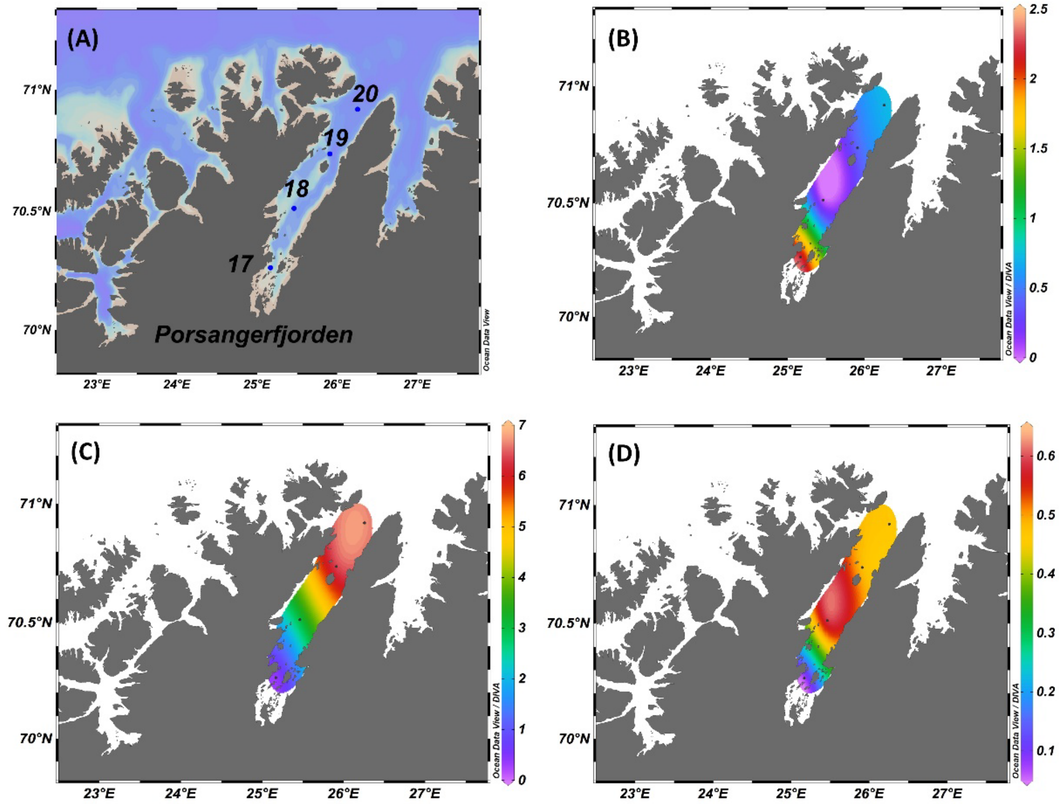

Fig. S4. Map of the study area. Stations sampled in Porsangerfjorden (A) showing spatial distribution of extracted chl *a* (µg L^{-1}) (B); total inorganic nitrogen (TIN) (µmol L^{-1}) (C); and inorganic phosphate ($PO4^{-3}$) (µmol L^{-1}) (D) at the chlorophyll maximum layer.

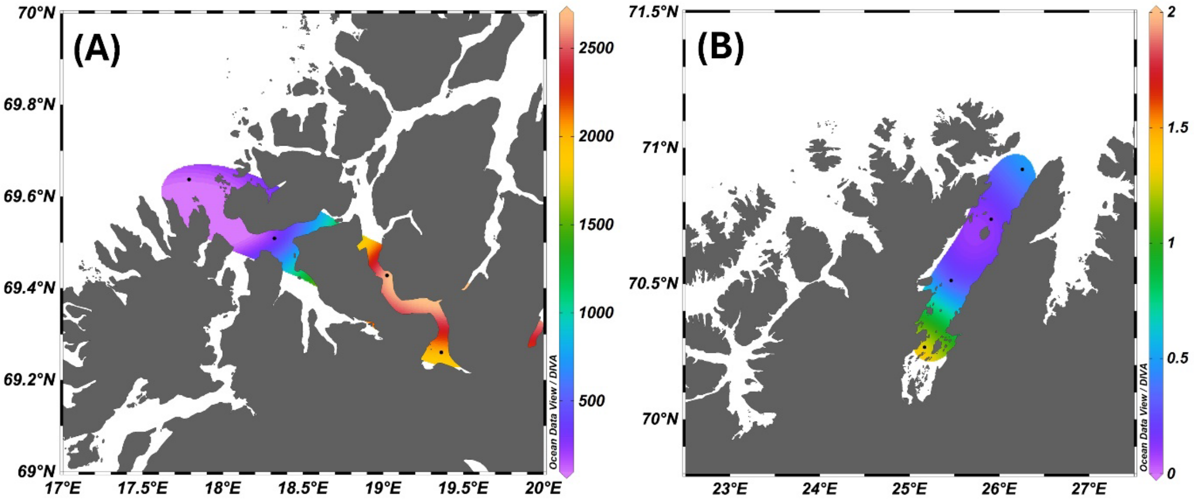

Fig. S5. Map of Balsfjorden (A) and Porsangerfjorden (B) showing the horizonal spatial distribution of *Chrysochromulina leadbeateri* cell densities (x 10^4 L^{-1}) at the chlorophyll maximum layer.

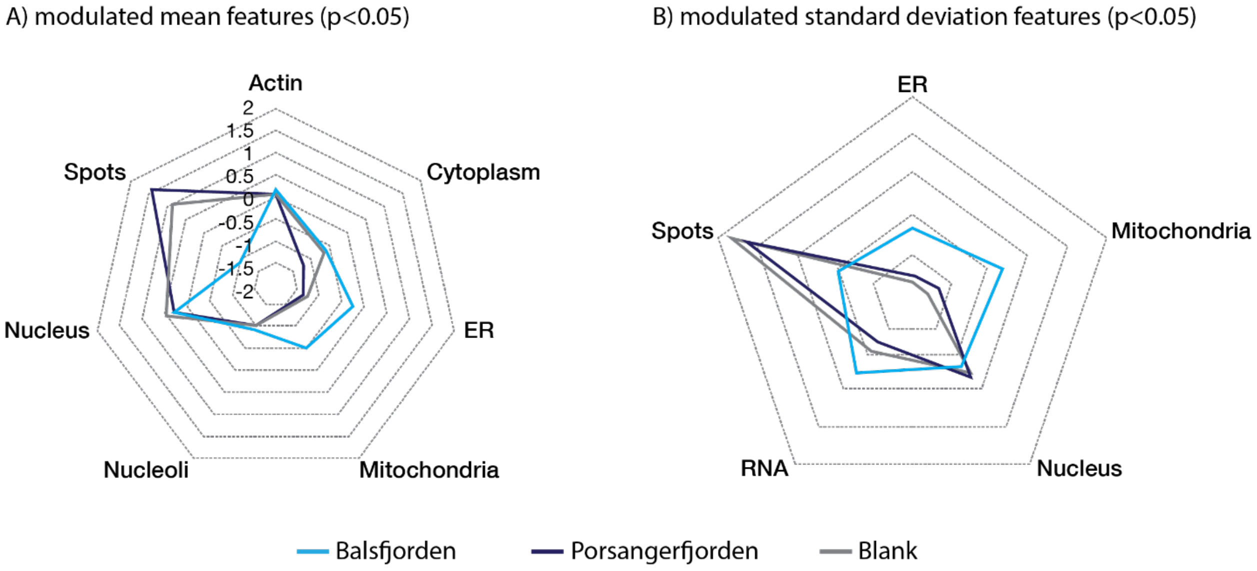

Fig. S6.

Network of High-Content-Screening (HCS) cytotoxicity assay of plankton cell samples based on affected organelles of the human osteosarcoma U-2 OS cell line. Differentially modulated factors were compared between *Chrysochromulina*-dominant bloom stations in Balsfjorden and non-bloom stations in Porsangerfjorden, and with respect to negative controls (non-parametric ANOVA, Kruskal Wallis test). Specific changes in effect on actin, cytoplasm, endoplasmic reticulum (ER), mitochondria, nucleoli, and the nucleus are indicated. Small dot-like structures in the cell correspond to protein or mRNA clusters (noted as "Spots" in the figure). Modulation response of affected organelles is with reference to: A) mean per well values ($p<0.05$); B) standard deviation ($p<0.05$). Figure shows a potent effect of Balsfjorden extracts compared to Porsangerfjorden samples, which yielded values very close to the blank.

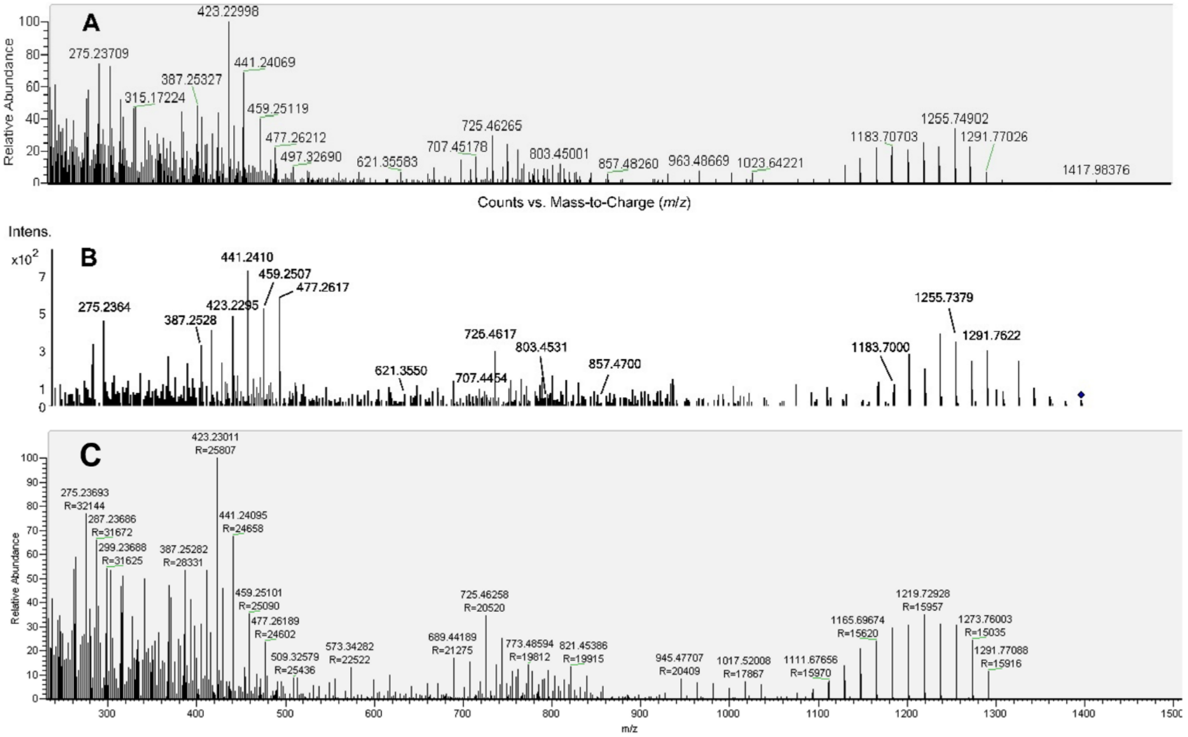

Fig. S7.

Mass spectrometry spectra of leadbeaterin-1 (LBT-1): A. (Q-TOF)MS/MS spectrum of the LBT-1 $[M + H]^+$ ion at *m/z* 1399.8338 detected in solid-phase extracts (SPE) of seawater from Balsfjorden (St 10); **B**. (Q-TOF)MS/MS spectrum of the LBT-1 $[M + H]^+$ ion at *m/z* 1399.8333 from methanolic cell extracts of *C. leadbeateri* strain UIO 394 (Norwegian Culture Collection of Algae, NORCCA). C. (QExactive)MS/MS spectrum of the LBT-1 $[M + H]^+$ ion at *m/z* 1399.83252 detected in solid-phase extracts (SPE) of seawater from Balsfjorden (St 09). Both mass spectrometry platforms produce comparable fragmentation patterns and daughter ions and compare closely with reference LBT-1 from cultured *C. leadbeateri* from NORCCA.

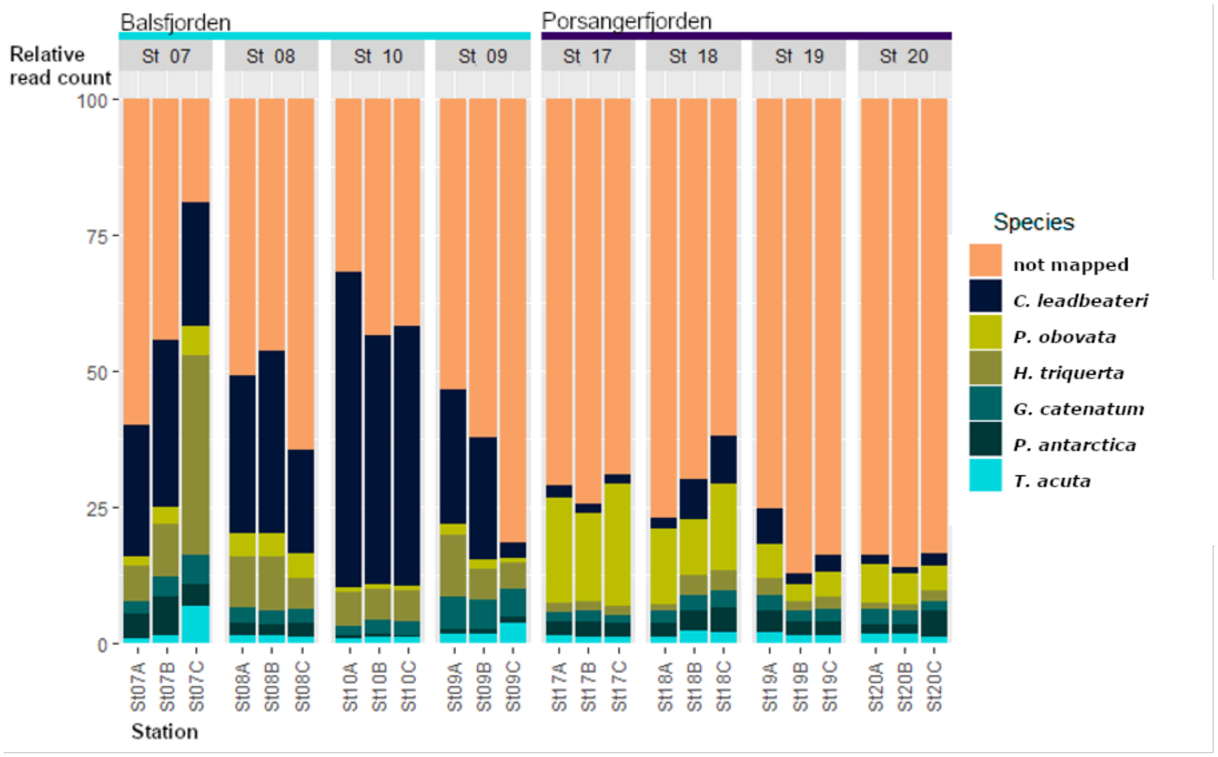

Fig. S8.

Sequence read mapping of the metatranscriptome to the reference transcriptomes. The stacked histograms show the relative proportion of sequenced RNA transcriptome read counts of the metatranscriptome that mapped to reference transcriptomes: *Chrysochromulina leadbeateri* (NORCAA strains UIO035 and UIO393); prasinophyte *Pyramimonas obovata*; dinoflagellates *Heterocapsa triquetra and Gymnodinium catenatum*; haptophyte *Phaeocystis antarctica* (all obtained from MMETSP); cryptophyte *Teleaulax acuta* (obtained from (*65*)); Balsfjorden, St 7–10 (light blue); Porsangerfjorden, St 17–20 (purple). The stations in Balsfjorden were reordered to correspond to the geographical location proximity; samples taken in triplicate (labelled A, B, C) are from different Niskin bottles deployed at the same depth.

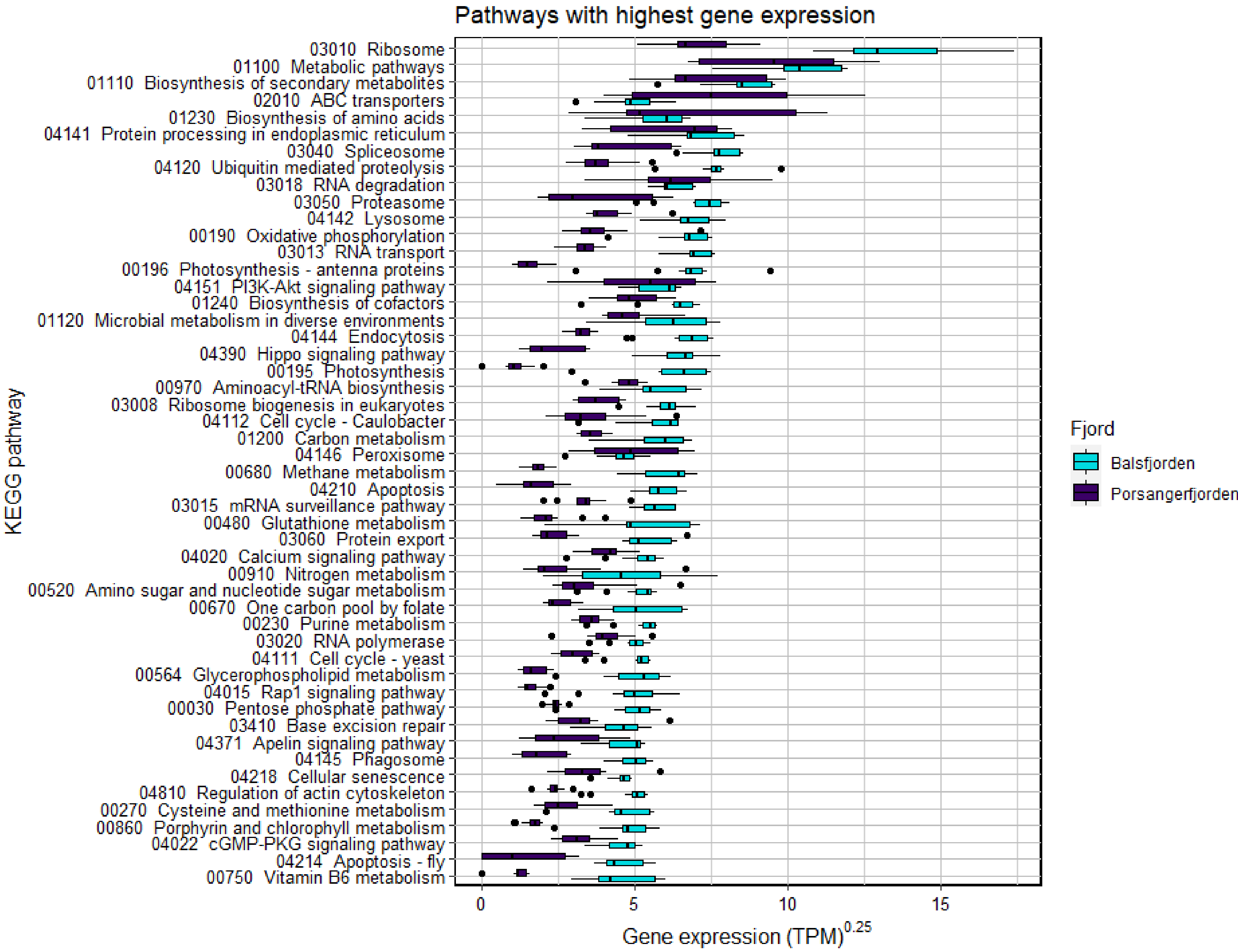

Fig. S9.

Gene expression of KEGG pathways. The 50 highest expressed KEGG pathways (Level 3) in Balsfjorden (light blue) and Porsangerfjorden (dark purple). Boxplots show the sum of gene expression in transcripts per million (TPM)$^{0.25}$ for all samples obtained from each fjord.

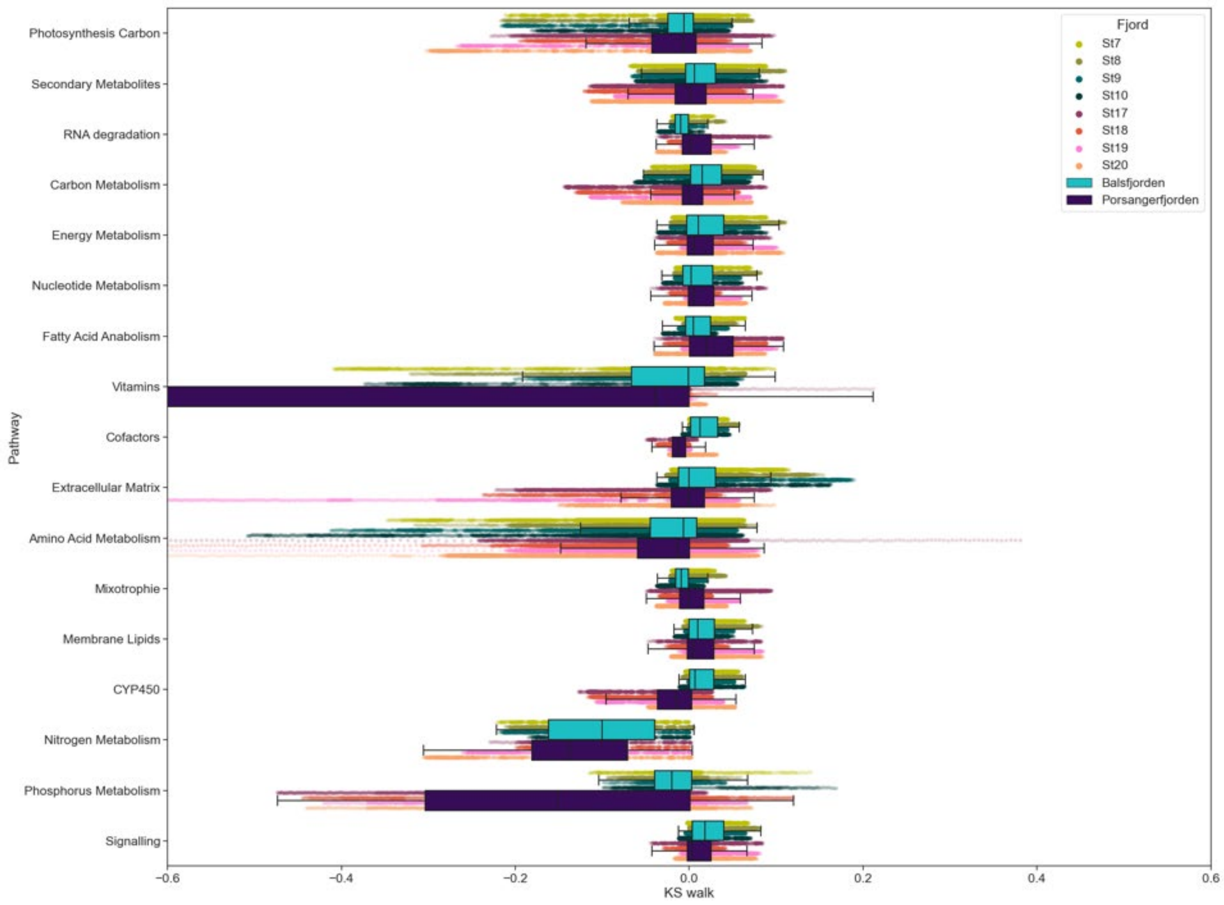

Fig. S10.

Gene Set Variation Analysis on metabolites (GSVAm). This GSVAm walk on mass difference networks (MDiN) was reconstructed based on the meta-metabolome. GSVAm enrichment scores are plotted by KEGG pathway, station, and fjord: Balsfjorden (light blue); Porsangerfjorden (dark purple). Positive values indicate enrichment towards higher log feature intensities; negative values indicate enrichment towards low feature intensities.

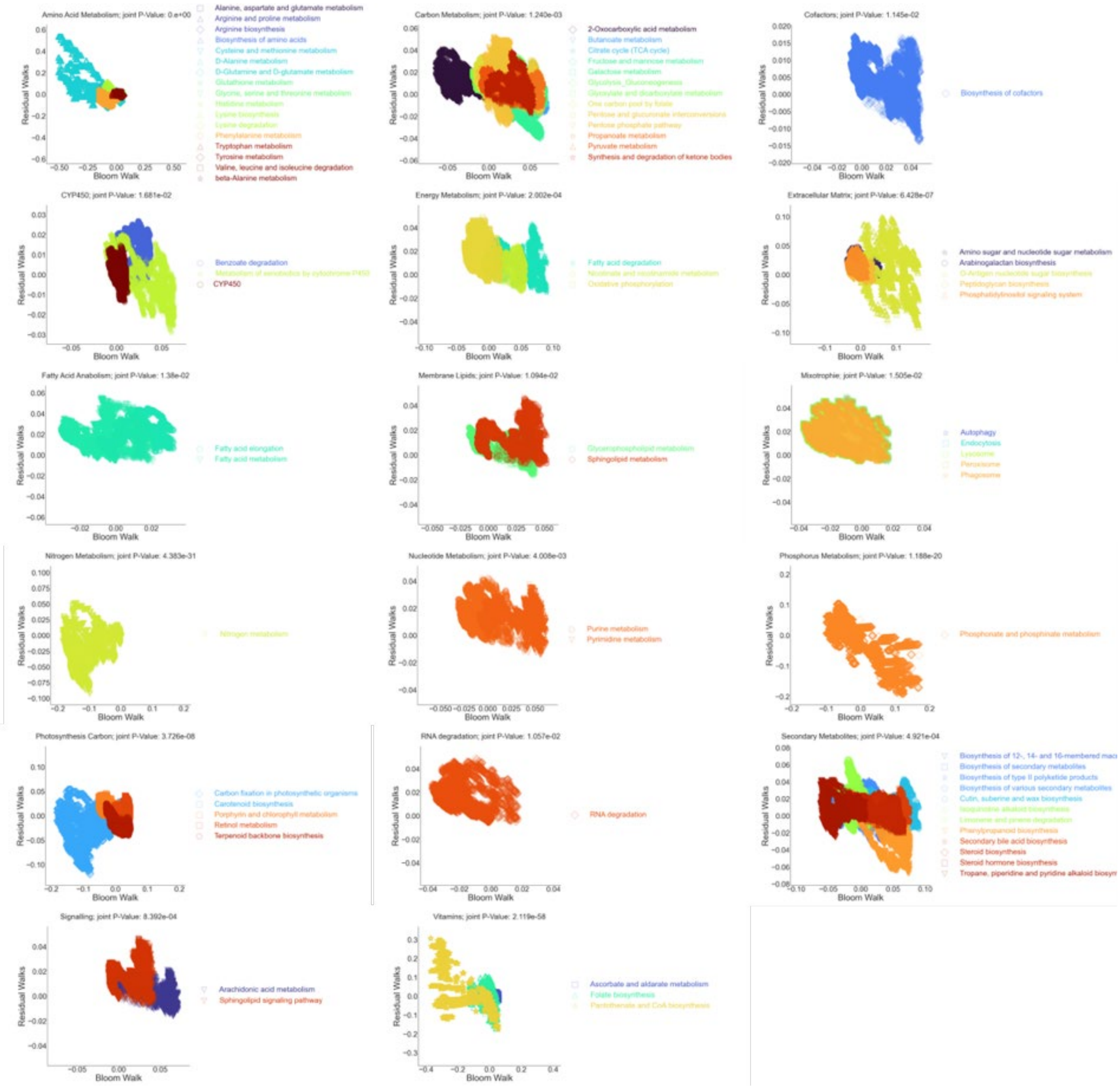

Fig. S11.

The 78 KEGG pathways are organized following 17 manually curated pathway categories. X-axes show the GSVAm walks of *Chrysochromulina* bloom-dominated St 10; y-axes indicate the residuals of non-bloom walks over the bloom walk. Quadrants 2 and 4 are of primary significance: Q2 indicates negative enrichment during the bloom and less negative (or even positive) enrichment in non-bloom conditions; Q4 indicates pathways that dominate during bloom conditions as compared to non-bloom. Significant features in Q1 and Q3 indicate those characteristic of Balsfjorden in general.

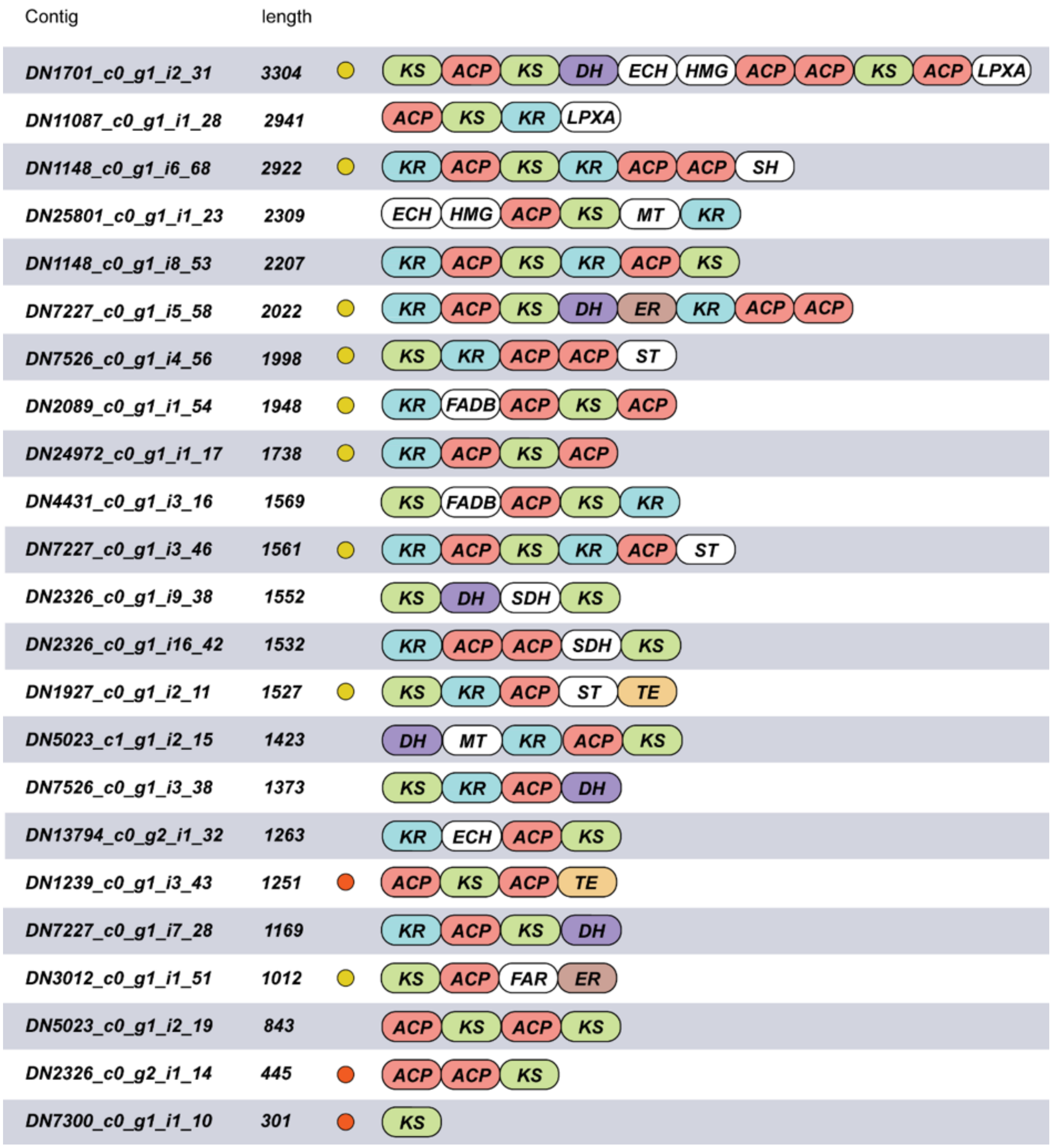

Fig. S12.

Domain compositions of upregulated PKS-like multi-enzymes among longest transcripts of *C. leadbeateri* strain UIO393. Transcripts upregulated during the bloom are marked with pale orange dots. Red dots highlight those with highest level of transcription.

Ketoacyl synthase (KS), ketoacyl reductase (KR), acyl carrier protein (ACP), enoyl-reductase (ER), dehydrogenase (DH), thioesterase (TE), methyltransferase (MT), sulfotransferase (ST), enoyl-CoA hydratase ECH, hydroxymethylglutaryl-coenzyme A synthase (HMG), LpxA-like fatty acid acyltransferase (LPXA), FAD/NAD(P)-dependent oxidoreductase (FADB), fatty acyl-coenzyme A reductases (FAR).

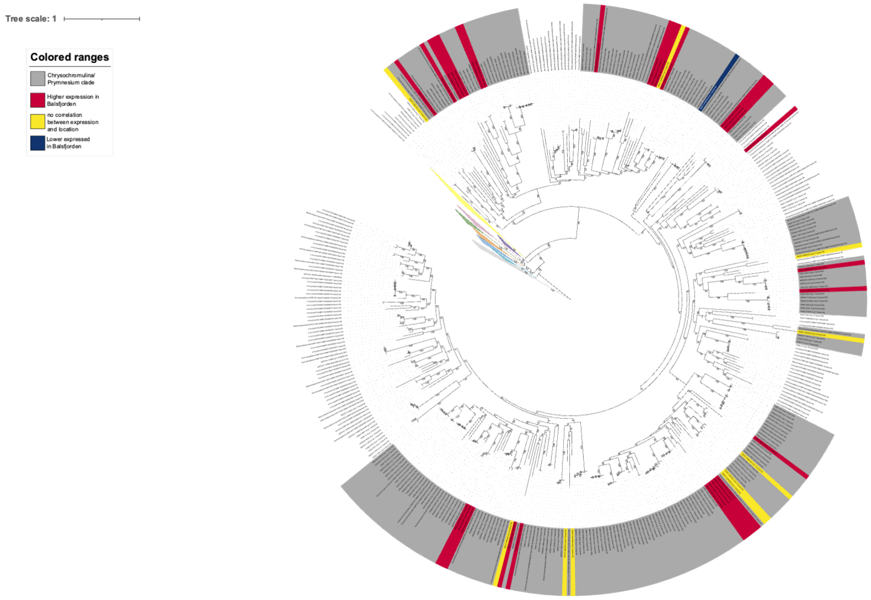

Fig. S13.

Phylogenetic tree of potential *C. leadbeateri* ketoacyl synthase (KS) domains aligned to other KS domains. At the root, phylogenetic outgroups are collapsed (triangles not to scale). Clusters that contained sequences from *Chrysochromulina* and *Prymnesium* species are indicated in grey. Sequences from this study are highlighted by color based on their expression in the study area (red: upregulated in Balsfjorden, blue: downregulated, yellow: no significance for location). Correlations were calculated as linear models (compare to Figure 5). Bootstrap values (100 replications) are indicated at the branches. ACP: Acyl carrier protein, FAS: fatty acid synthase, RPS: Non-ribosomal polyketide synthetase/synthase.

Table S1.

Environmental parameters and cruise information (station, event [ship station], date, time, geographical coordinates) across the fjord study area in northern Norway. *C. leadbeateri* cell densities are given for the key bloom stations in Balsfjorden (St 7 to 10) and non-bloom Porsangerfjorden (St 17 to 20). Temperature, salinity, silicate (SiO_4), inorganic nitrogen (NO_2^-, NO_3^-, and NH_4^+), total N, PO_4^{-3}, N:P ratios, and particulate organic nutrients (C,N,P) were determined at the chlorophyll maximum layer (depth estimated from *in situ* fluorescence profiles). For the full dataset, see research cruise HE533 on Pangea https://doi.org/10.1594/PANGAEA.903511.

See separate excel file for Table S1.

Table S2.

Fragmentation pattern of the putative leadbeaterin-1 (LBT-1) ion (*m/z* 1399.8338) in solid-phase extracts (SPE) of seawater from Balsfjorden (HE533, St 9) for the *QExactive* and *Q-TOF* mass spectrometric platforms.

Elemental Composition	Theoretical Mass	*m/z* observed *QExactive*	Intensity *QExactive*	Δ (ppm) *QExactive*	*m/z* observed *Q-TOF*	Intensity *Q-TOF*	Δ (ppm) *Q-TOF*
C67 H128 O27 Cl	1399.83260	1399.83252*	26513838*	-0.06*	1399.8333*	211999*	-0.5*
C67 H116 O21 Cl	1291.76921	1291.76973	70478	0.40	1291.76760	152.84	1.25
C67 H114 O20 Cl	1273.75865	1273.75937	167286	0.57	1273.76520	217.01	-5.14
C67 H112 O19 Cl	1255.74809	1255.74882	208972	0.59	1255.74770	472.52	0.31
C67 H110 O18 Cl	1237.73752	1237.73873	217059	0.98	1237.73830	268.64	-0.63
C67 H108 O17 Cl	1219.72696	1219.72916	222783	1.81	1219.71620	145.20	8.82
C67 H106 O16 Cl	1201.71639	1201.71569	204958	-0.59			
C67 H104 O15 Cl	1183.70583	1183.70639	205346	0.48	1183.70100	372.05	4.08
C67 H102 O14 Cl	1165.69526	1165.69579	164393	0.45	1165.68980	255.85	4.68
C67 H100 O13 Cl	1147.68470	1147.68557	141790	0.76			
C57 H81 O18	1053.54174	1053.54197	5138	0.22			
C57 H79 O17	1035.53118	1035.53095	37919	-0.22			
C57 H77 O16	1017.52061	1017.51996	42890	-0.64	1017.51340	173.51	7.09
C57 H75 O15	999.51005	999.50963	27299	-0.42			
C57 H73 O14	981.49948	981.49874	40747	-0.76			
C57 H71 O13	963.48892	963.48754	46721	-1.43			
C57 H69 O12	945.47835	945.47712	50820	-1.31			
C57 H67 O11	927.46779	927.46766	19797	-0.14			
C43 H69 O11	761.48344	761.48419	90292	0.99			
C43 H67 O10	743.47287	743.47365	169597	1.05			
C43 H65 O9	725.46231	725.46267	226374	0.49			
C24 H42 O7 Cl	477.26136	477.26198	154225	1.30	477.26100	301.81	0.75
C24 H40 O6 Cl	459.25079	459.25110	238860	0.66	459.25080	317.97	-0.02
C24 H38 O5 Cl	441.24023	441.24085	478456	1.40	441.24170	434.72	-3.33
C24 H36 O4 Cl	423.22966	423.23012	680175	1.08	423.22880	296.55	2.03
C24 H34 O3 Cl	405.21910	405.21992	206959	2.02			
C29 H41 O4	453.29994	453.30025	84768	0.69			
C24 H35 O4	387.25299	387.25284	356919	-0.39	387.25210	147.37	2.30
C19 H30 O3 Cl	341.18780	341.18835	330680	1.60			
C21 H31 O	299.23694	299.23689	367751	-0.17			
C19 H31 O	275.23694	275.23694	512075	-0.01	275.23550	234.43	5.23

Table S3.

Theoretical and observed values of putative LBT analogs detected in solid-phase extracts (SPE) of seawater from Balsfjorden (HE533, St 9) analyzed by the QExactive and Q-TOF mass spectrometric platforms.

**Elemental composition (M+H)**	**Theoretical mass**	*m/z* observed	Intensity	Δ (ppm)	Retention (min)	*m/z* observed	Intensity	Δ (ppm)	Retention (min)
		Orbitrap	*Orbitrap*	*Orbitrap*	*Orbitrap*	*Q-TOF*	*Q-TOF*	*Q-TOF*	*Q-TOF*
**C67 H125 O27 Cl2**	**1431.77798**	1431.77795*	17708370*	-0.02*	10.02	1431.7758*	110513*	-1.5*	5.36
**C67 H128 O27 Cl**	**1399.83260**	1399.83252*	26513838*	-0.06*	10.10	1399.8333*	211999*	-0.5*	5.44
**C67 H129 O27**	**1365.87157**	1365.87305*	3867258*	1.08*	9.98	1365.8702*	24922*	-1.00*	5.65
**C67 H128 O30 Cl S**	**1479.78941**	1479.78845*	1477823*	-0.65*	10.14	1479.7904*	9431*	-0.70*	5.33
**C67 H126 O27 Cl**	**1397.81695**	1397.81958*	680363*	-1.88*	9.90	1397.8109*	15865*	-4.3*	5.27
**C69 H130 O29 Cl**	**1457.83808**	1457.83850*	662345*	0.29*	10.15	1457.8325*	31230*	-3.80	5.49

Table S4.

KEGG pathways included in categories “Cell growth and death” and “Mixotrophy in Fig. 2. Based on the Kyoto Encyclopedia of Genes and Genomes (KEGG) https://www.genome.jp/kegg/

Genes included in “Cell growth and death” were annotated as the following KEGG pathways:	Genes included in “Mixotrophy” were annotated as the following KEGG pathways:
04110 Cell cycle	04144 Endocytosis
04111 Cell cycle – yeast	04145 Phagosome
04112 Cell cycle – Caulobacter	04142 Lysosome
04113 Meiosis – yeast	04140 Autophagy – animal
04114 Oocyte meiosis	04138 Autophagy – yeast
04115 p53 signaling pathway	04136 Autophagy – other
04210 Apoptosis	
04214 Apoptosis – fly	
04216 Ferroptosis	
04217 Necroptosis	
04218 Cellular senescence	

Table S5.

p-values (_p) and estimates (_e) for linear models correlating gene expression (z-scores) and environmental parameters. NP: inorganic N:P ratio, lon: longitude, sal: salinity, Si: dissolved silicate ($Si0_4$), sm: shannon-index-micro-fraction, sn: shannon-nano, sp: shannon-pico, em: evenness-micro-fraction, en: evenness-nano, ep: evenness-pico

See separate excel file.

Table S6.

Diversity and Evenness calculated from 18S rRNA gene metabarcoding at Balsfjorden and Porsangerfjorden stations. Values were separately calculated for operationally defined micro- (>20 μm), nano- (3-20μm), and pico-plankton (0.2-3 μm) size-fractions.

Station	Shannon.micro	Shannon.nano	Shannon.pico	Eveness.micro	Eveness.nano	Eveness.pico
7	3.80998385	4.01182136	4.30054287	0.65597286	0.59393879	0.70706808
8	3.1414426	4.6160658	4.06606349	0.56002467	0.6809577	0.66952637
9	2.17775154	3.54967268	2.77590855	0.41546292	0.55221135	0.49551047
10	3.27699181	2.68700427	2.4531273	0.57452959	0.42379369	0.45405771
17	3.35262108	4.04145946	3.59572907	0.61079598	0.62353026	0.59298424
18	3.8861367	4.34782915	2.84789511	0.68415821	0.65836858	0.48115327
19	4.26898229	4.31409802	2.93570312	0.7202692	0.65543627	0.50838634
20	3.49760605	3.96578633	4.21054511	0.61004672	0.60888166	0.68269702

Table S7.

Co-expression enrichment analysis. Genes with similar expression patterns as PKS contigs were identified with the command genefinder from the R package ‘genefilter’*(67)*, using the settings method= "euc", scale= "none". A distance cutoff value of 1.0 was chosen, to eliminate genes beyond that distance. An enrichment analysis was done to see whether pathways were more prominent in a cluster than statistically expected. The "fold enrichment" was calculated as (x/k)/(m/N) , x being the number of genes in the selected pathway in the cluster, k being the number of genes in the cluster in total, m being the total number of genes in the selected pathway in the whole data set and N being the total number of annotated genes to any pathway. The p-value for enrichment was calculated using the command phyper(q=x -1, m=m, n=n, k=k, lower.tail=FALSE). A p-value < 0.05 was considered statistically significant.

Contig	Enriched pathway (KEGG level 3)		p-value
DN7300_c0_g1_i2	01241	Biosynthesis of cofactors	0.02256
DN2089_c0_g1_i1	04024	cAMP signaling pathway	0.00382
	04111	Cell cycle	0.01293
DN41702_c0_g1_i1	04111	Cell cycle	0.00495
DN24972_c0_g1_i1	00270	Cysteine and methionine metabolism	0.02812
	04024	cAMP signaling pathway	0.00591
	04111	Cell cycle	0.01735
